# Supplementary material for: Functional interactions between posttranslationally modified amino acids of methyl-coenzyme M reductase in Methanosarcina acetivorans
Source: PLoS Biol. 2020 Feb 24;18(2):e3000507. doi: 10.1371/journal.pbio.3000507 (PMC7058361; doi:10.1371/journal.pbio.3000507)

A

Ion	Calculated mass (Da)	Observed mass (Da)	Error (ppm)
$b4^{+}$	465.2497	465.2489	1.76
$b6^{+}$	685.3167	685.3154	1.93
$b8^{+}$	913.4277	913.4268	1.05
$b12^{+}$	1385.5654	1385.5629	1.75
$b13^{+}$	1442.5868	1442.5833	2.47
$b14^{+}$	1513.6239	1513.6209	1.99
$b15^{+}$	1614.6716	1614.6682	2.10
$b16^{+}$	1728.7145	1728.7113	1.85
$b17^{+}$	1827.7829	1827.7798	1.75
$b17^{2+}$	914.3951	914.3932	2.14
$b18^{+}$	1940.8670	1940.8634	1.89
$b26^{2+}$	1395.6124	1395.6097	1.96
$y3^{+}$	417.2457	417.2450	1.53
$y5^{+}$	629.3254	629.3242	1.88
$y6^{+}$	742.4094	742.4081	1.75
$y7^{+}$	799.4309	799.4293	2.05
$y8^{+}$	928.4735	928.4714	2.24
$y9^{+}$	1043.5004	1043.4977	2.58
$y10^{+}$	1100.5219	1100.5199	1.85
$y11^{+}$	1228.5805	1228.5778	2.18
$y11^{2+}$	614.7939	614.7929	1.66
$y12^{+}$	1391.6438	1391.6412	1.87
$y13^{+}$	1478.6758	1478.6726	2.16
$y13^{2+}$	739.8416	739.8403	1.76
$y14^{+}$	1591.7599	1591.7570	1.82
$y14^{2+}$	796.3836	796.3824	1.46
$y15^{+}$	1690.8283	1690.8238	2.69
$y15^{2+}$	845.9178	845.9156	2.61
$y16^{+}$	1804.8712	1804.8651	3.40
$y16^{2+}$	902.9393	902.9363	3.33
$y17^{+}$	1905.9189	1905.9162	1.41
$y17^{2+}$	953.4631	953.4615	1.71
$y19^{2+}$	1017.4924	1017.4910	1.39
$y23^{2+}$	1253.5612	1253.5574	3.06
$y24^{2+}$	1310.1033	1310.1031	0.10
$y25^{2+}$	1367.6167	1367.6140	2.03

B

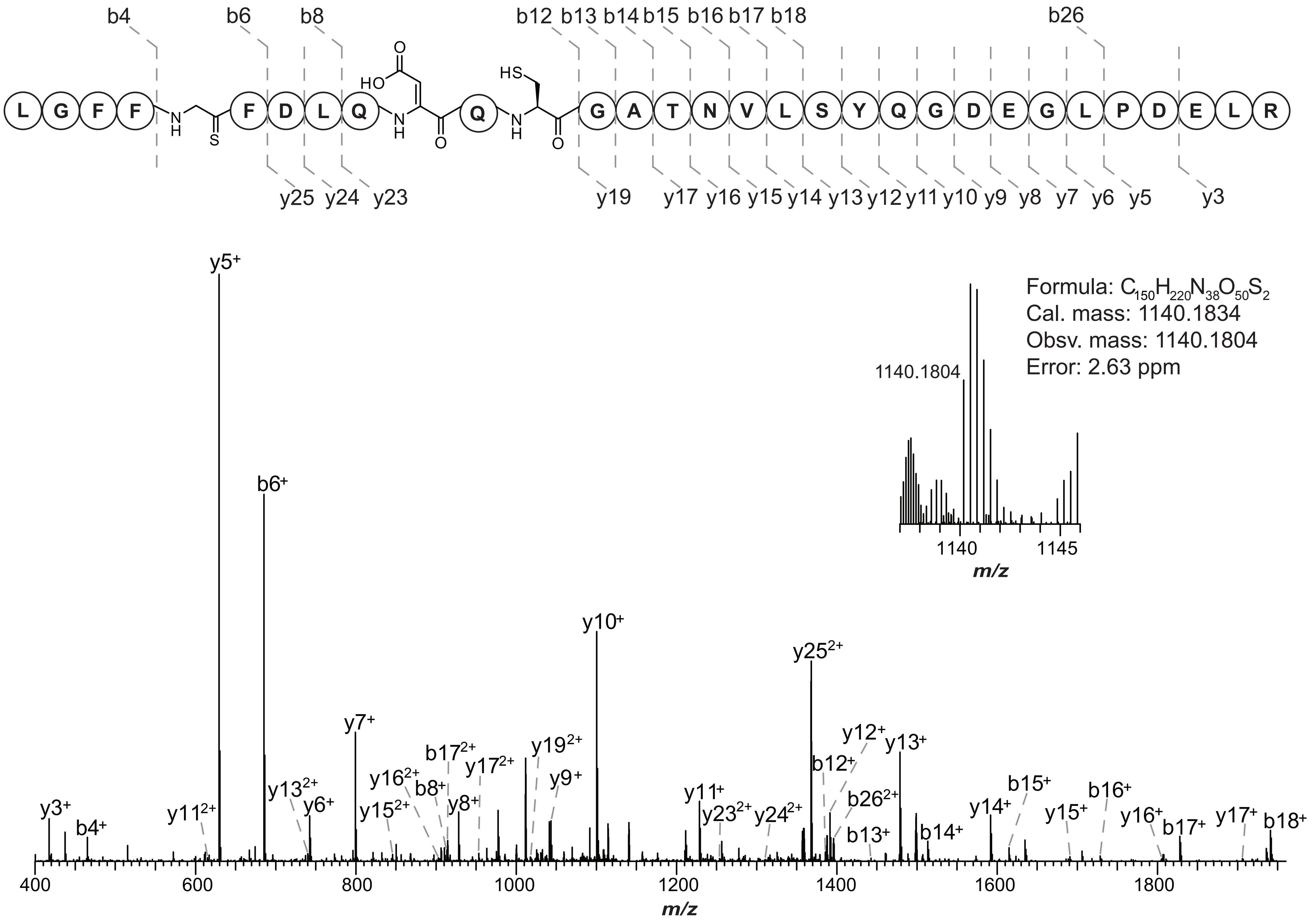

Supplement: S1 Fig — (A) The 1140.18-Da molecular ion was subjected to CID with assigned ions indicated in tabular form. (B) The triply charged molecular ion shows the presence of a thioamide and absence of the methylcysteine (1140.18 Da). MS/MS spectral data indicate that the thioamide is located at the Gly465 (b4 and b6) and that there is no methylation on Cys472 (b12 and y23). Equivalent data were obtained with strain ΔmamAΔmcmA. CID, collision-induced dissociation; HR-ESI MS/MS, high-resolution electrospray ionization tandem mass spectrometry; mamA, methylarginine modification; mcmA, methylcysteine modification; MCR, methyl-coenzyme M reductase; MS, mass spectrometry. (PDF) [file pbio.3000507.s001.pdf]
