# Supplementary figures and images for: Functional interactions between posttranslationally modified amino acids of methyl-coenzyme M reductase in Methanosarcina acetivorans
Source: PLoS Biol. 2020 Feb 24;18(2):e3000507. doi: 10.1371/journal.pbio.3000507 (PMC7058361; doi:10.1371/journal.pbio.3000507)

**A** <sup>271</sup>**H**AALVSMGEMLPAR<sup>284</sup>

1496 Da

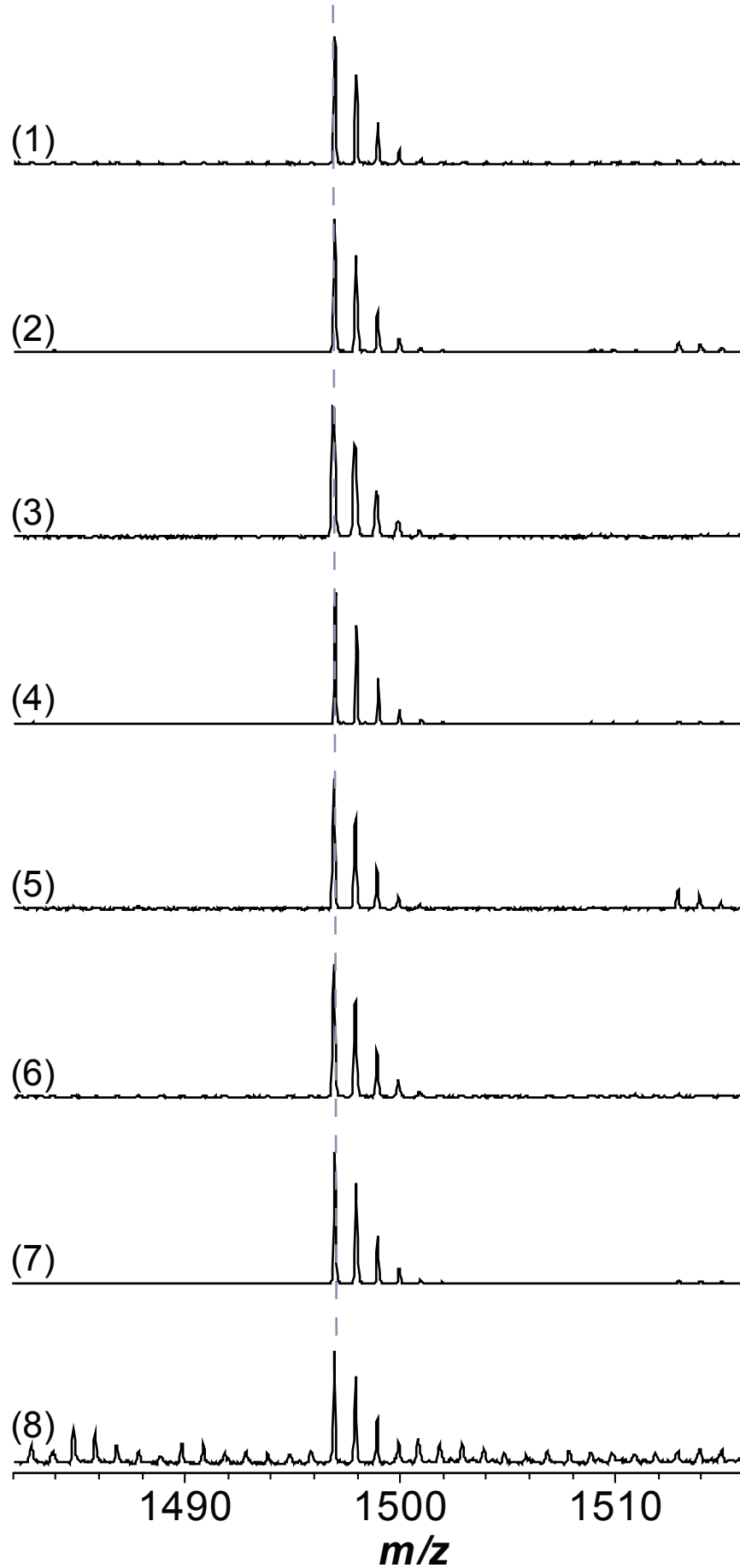

**B** <sup>408</sup>FPTALEDHFGG**SQ**R<sup>421</sup>

1561 Da

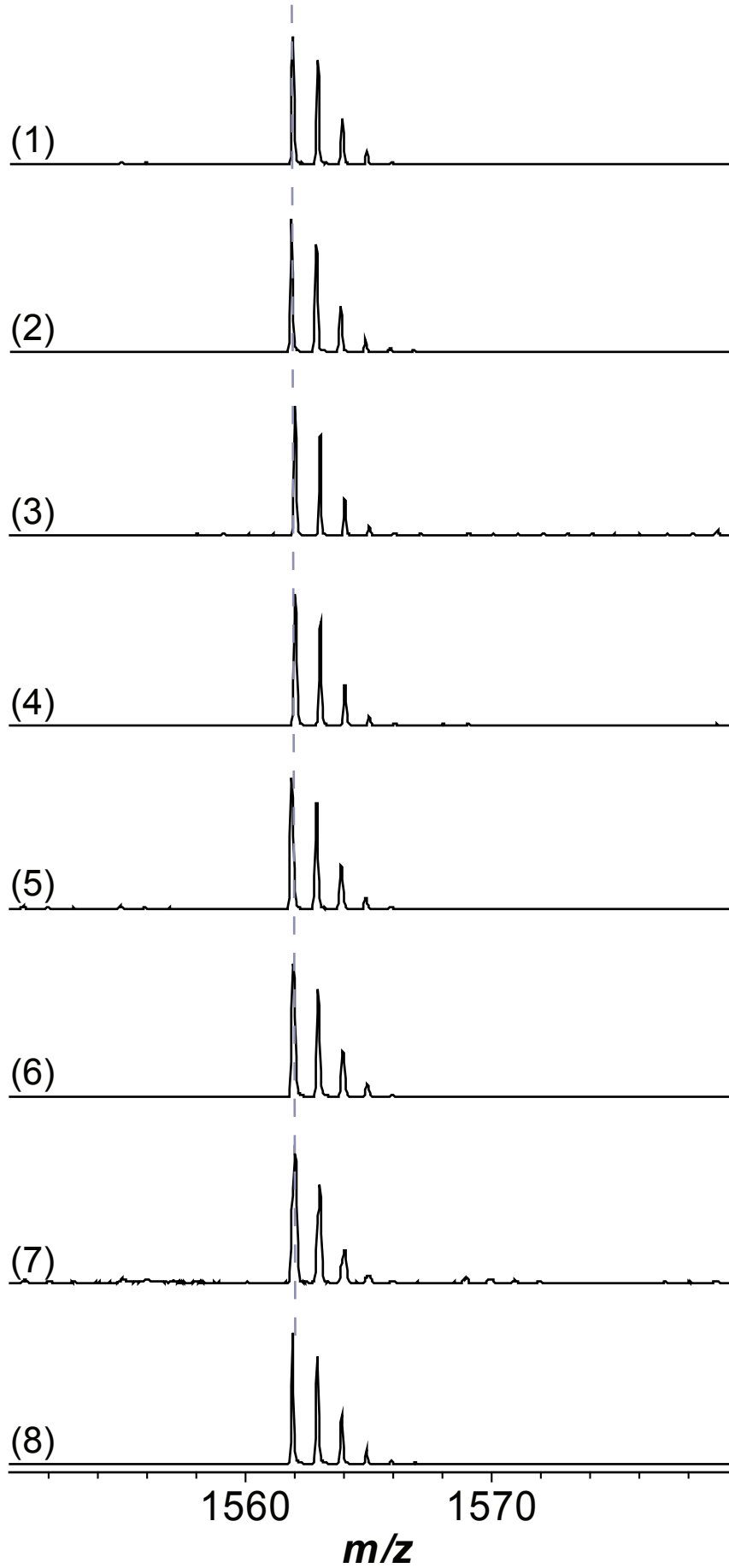

Supplement: S2 Fig — (A) Spectrum obtained from trypsinolysis of MCR obtained from WT (WWM60) and mutants lacking ycaO-tfuA, mcmA, and mamA in all possible combinations. The H271-R284 peptide contains His271 (red) that is modified to 3-methyhistidine. (B) Spectrum obtained from trypsinolysis of MCR from strains mentioned above. The F408-R421 peptide contains Gln420 (red) that is unmodified in M. acetivorans. Individual spectra are labeled with numbers in parentheses as indicated in Fig 3A. MALDI-TOF-MS, matrix-assisted laser desorption/ionization time-of-flight mass spectrometry; mamA, methylarginine modification; mcmA, methylcysteine modification; MCR, methyl-coenzyme M reductase; McrA, alpha subunit of MCR; WT, wild-type. (PDF) [file pbio.3000507.s002.pdf]

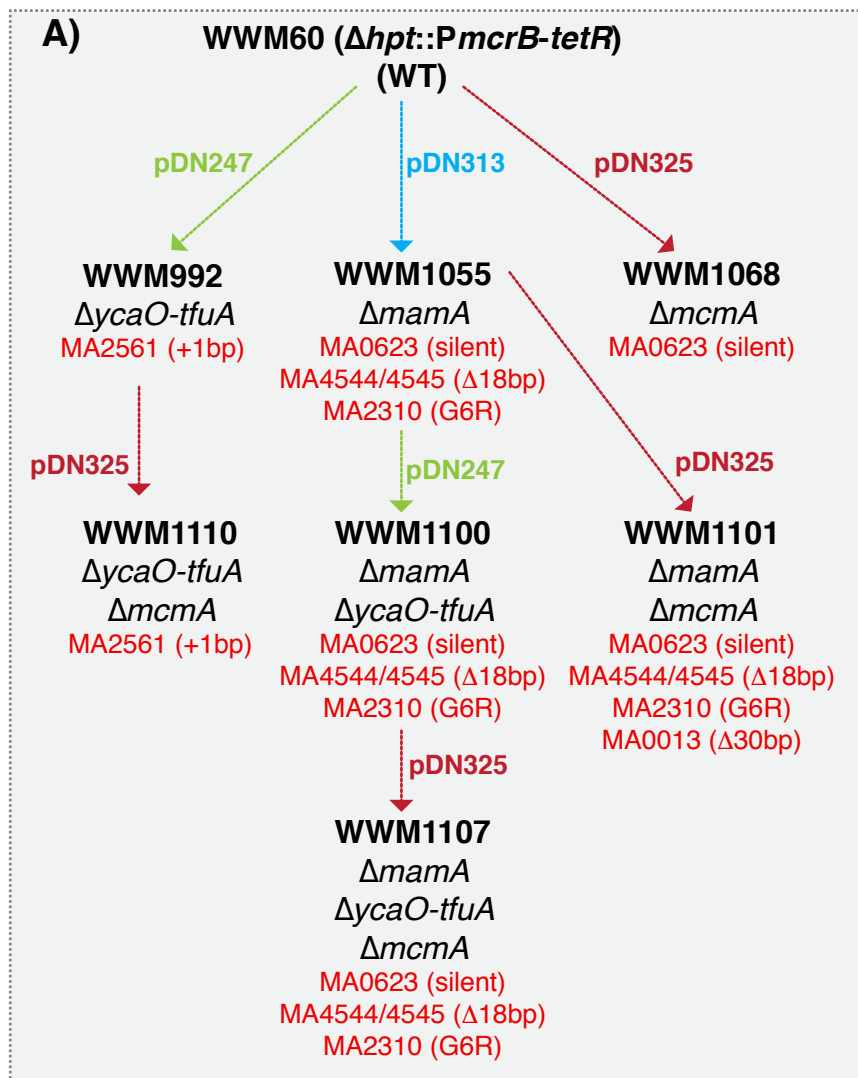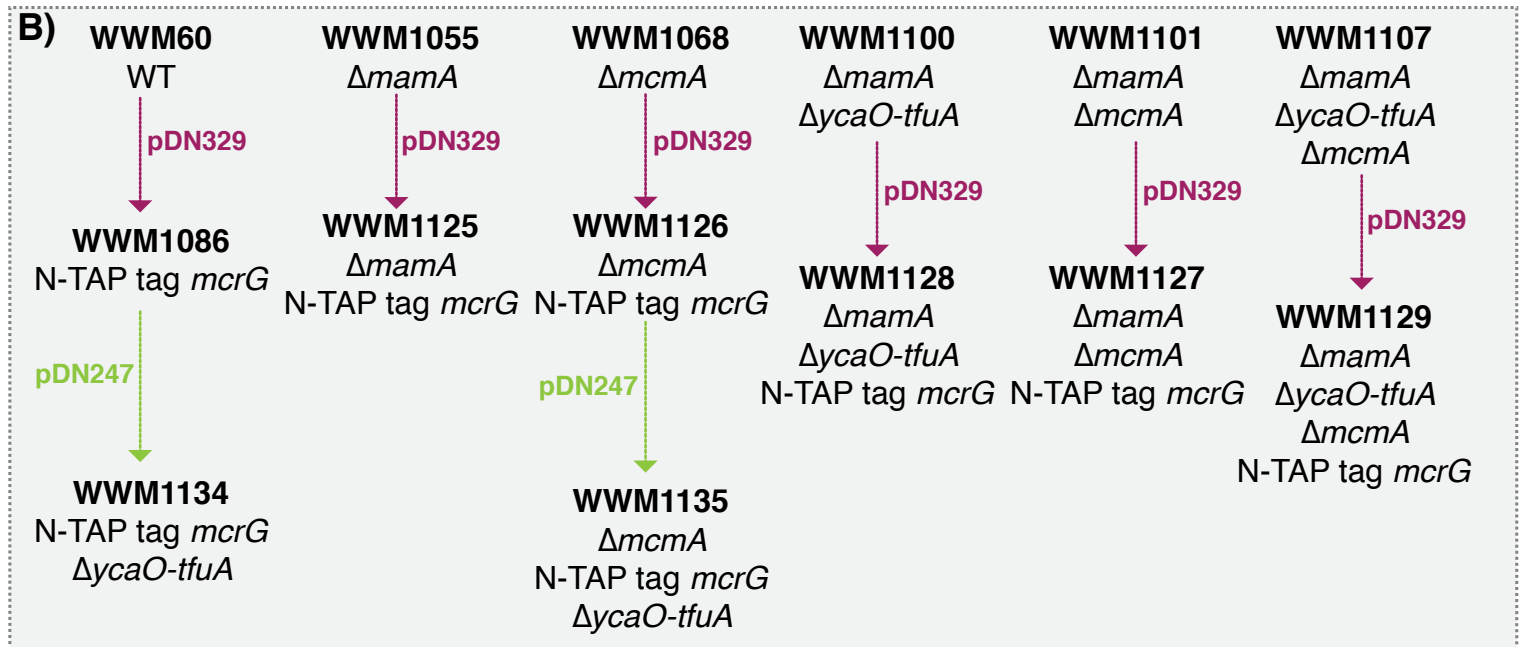

Supplement: S3 Fig — (A) A schematic outlining the order in which ycaO-tfuA, mamA, and mcmA were deleted to generate single-, double-, and triple-deletion mutants using the corresponding Cas9-based gene-editing vectors (pDN247 for ΔycaO-tfuA in green; pDN313 for ΔmamA in blue; pDN325 for ΔmcmA in red). Mutations in each strain are noted in red and were identified using whole-genome resequencing. (B) A schematic outlining the order in which an N-terminal TAP tag was introduced at the mcrG locus in WWM60 (WT) as well as strains lacking ycaO-tfuA, mamA, and mcmA in all possible combinations using the Cas9-based gene-editing vector pDN329 (in red). We were unable to introduce the TAP-tag at the N-terminus of mcrG in WWM992 or WW1110 using pDN329; however, it was possible to delete the ycaO-tfuA locus in WWM1086 and WWM1126 using the Cas9-based gene-editing vector pDN247 (in green). mamA, methylarginine modification; mcmA, methylcysteine modification; mcrG, allele encoding the gamma subunit of MCR; TAP, tandem-affinity purification; WT, wild-type. (PDF) [file pbio.3000507.s003.pdf]

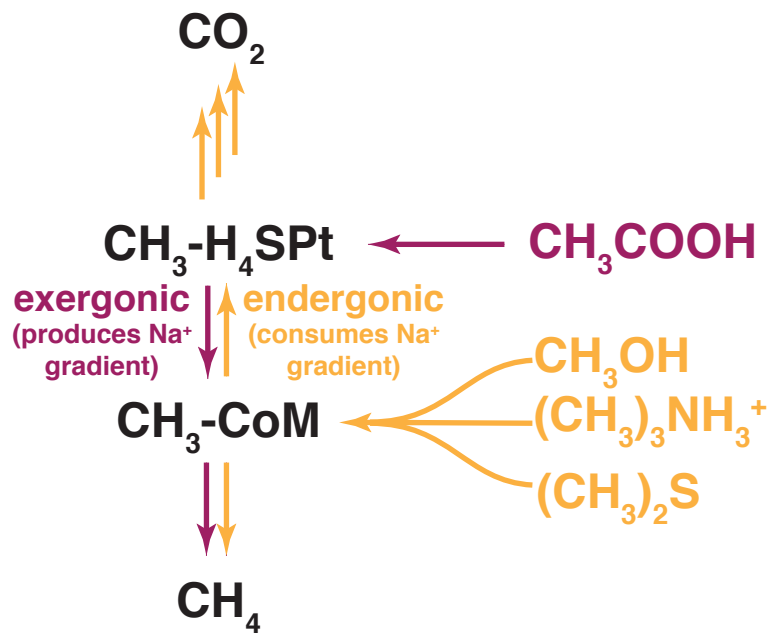

Supplement: S9 Fig — Methyl-transfer reactions from methylotrophic substrates like methanol (CH3OH), TMA ([CH3]3NH3+), and DMS (CH3-S-CH3) lead to the generation of CoM (CH3-CoM), which is disproportionated to methane (CH4) and carbon dioxide (CO2; metabolic flux is shown as orange arrows). Notably, the first step in oxidation of CH3-CoM to CO2 is the energy-requiring transfer of the methyl moiety to generate methyl-tetrahydrosarcinapterin (CH3-H4SPt). In contrast, aceticlastic methanogenesis leads to the formation of CH3-H4SPt, followed by reduction to CH4 (red arrows). Thus, the second step of the pathway is exergonic. CoM, methyl-coenzyme M; DMS, dimethyl sulfide; TMA, trimethylamine. (PDF) [file pbio.3000507.s009.pdf]
