# Supplementary material for: Functional interactions between posttranslationally modified amino acids of methyl-coenzyme M reductase in Methanosarcina acetivorans
Source: PLoS Biol. 2020 Feb 24;18(2):e3000507. doi: 10.1371/journal.pbio.3000507 (PMC7058361; doi:10.1371/journal.pbio.3000507)

A

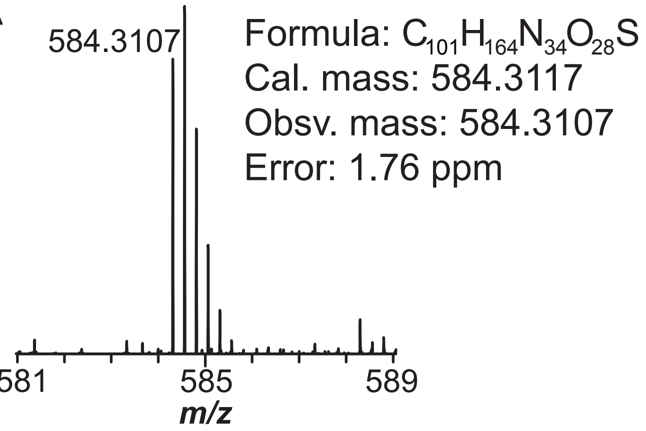

B

Ion	Calculated mass (Da)	Observed mass (Da)	Error (ppm)
$b5^+$	569.3229	569.3217	2.09
$b6^+$	739.4396	739.4379	2.39
$b8^+$	966.5778	966.5763	1.64
$b9^+$	1023.5993	1023.5974	1.88
$b11^+$	1234.6950	1234.6919	2.49
$b12^+$	1363.7376	1363.7342	2.48
$b21^{2+}$	1115.0948	1115.0925	2.03
$y6^+$	647.3148	647.3138	1.59
$y8^+$	817.4203	817.4191	1.53
$y10^+$	971.4946	971.4923	2.31
$y11^+$	1100.5371	1100.5344	2.49
$y13^+$	1311.6328	1311.6299	2.24
$y14^+$	1368.6543	1368.6507	2.64
$y15^+$	1524.7554	1524.7528	1.72
$y16^+$	1595.7925	1595.7880	2.86
$y17^+$	1765.9093	1765.9019	4.20
$y20^{2+}$	1045.5538	1045.5522	1.52

C

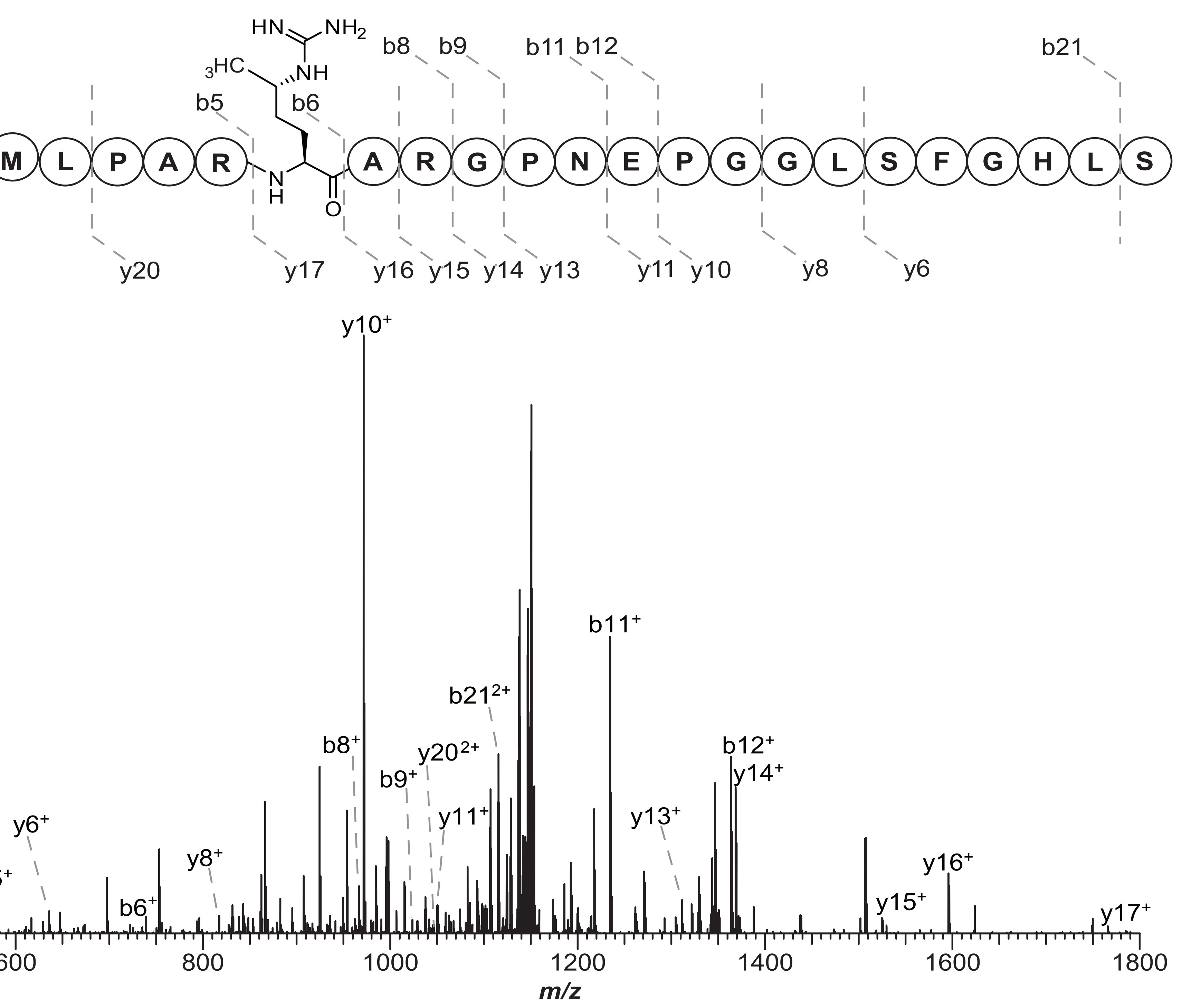

Supplement: S5 Fig — (A) The 4+ molecular ion shows the presence of a methylation (584.31 Da). (B) The 584.31-Da ion was subjected to CID with assigned ions indicated in tabular form. (C) MS/MS spectral data locate the methylation to Arg285 (b6 and y17). Equivalent data were obtained with strains ΔycaO-tfuA, ΔmcmA, and ΔmcmAΔycaO-tfuA. CID, collision-induced dissociation; HR-ESI MS/MS, high-resolution electrospray ionization tandem mass spectrometry; mcmA, methylcysteine modification; MCR, methyl-coenzyme M reductase; MS, mass spectrometry. (PDF) [file pbio.3000507.s005.pdf]
