# Supplementary material for: Functional interactions between posttranslationally modified amino acids of methyl-coenzyme M reductase in Methanosarcina acetivorans
Source: PLoS Biol. 2020 Feb 24;18(2):e3000507. doi: 10.1371/journal.pbio.3000507 (PMC7058361; doi:10.1371/journal.pbio.3000507)

A

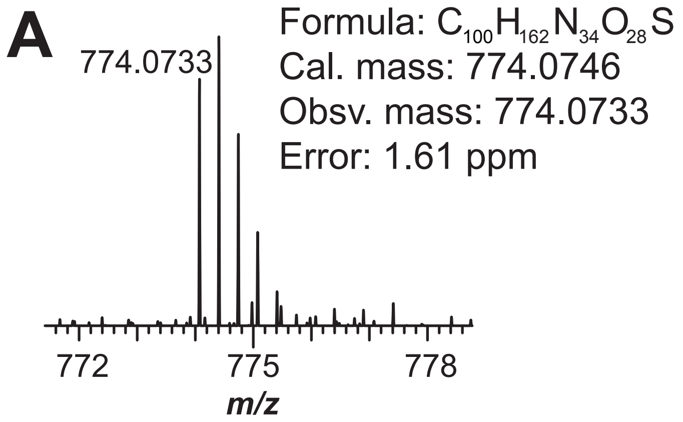

B

Ion	Calculated mass (Da)	Observed mass (Da)	Error (ppm)
$b5^{+}$	569.3229	569.3217	1.98
$b8^{2+}$	476.7848	476.7836	2.43
$b9^{2+}$	505.2955	505.2947	1.60
$b11^{2+}$	610.8433	610.8420	2.21
$b12^{2+}$	675.3646	675.3632	2.16
$b17^{2+}$	880.9705	880.9692	1.49
$b18^{2+}$	954.5047	954.5027	2.15
$b19^{2+}$	983.0155	983.0131	2.33
$b19^{3+}$	655.6794	655.6779	2.39
$b20^{2+}$	1051.5449	1051.5424	2.08
$b20^{3+}$	701.3657	701.3643	2.44
$b21^{3+}$	739.0604	739.0588	2.12
$y3^{+}$	356.1929	356.1920	2.47
$y4^{+}$	413.2144	413.2133	2.47
$y5^{+}$	560.2828	560.2814	2.46
$y10^{+}$	971.4946	971.4922	2.40
$y13^{+}$	1311.6328	1311.6291	2.82
$y14^{+}$	1368.6543	1368.6537	0.45
$y17^{2+}$	876.4505	876.4488	1.92
$y20^{2+}$	1038.5460	1038.5438	2.13

C

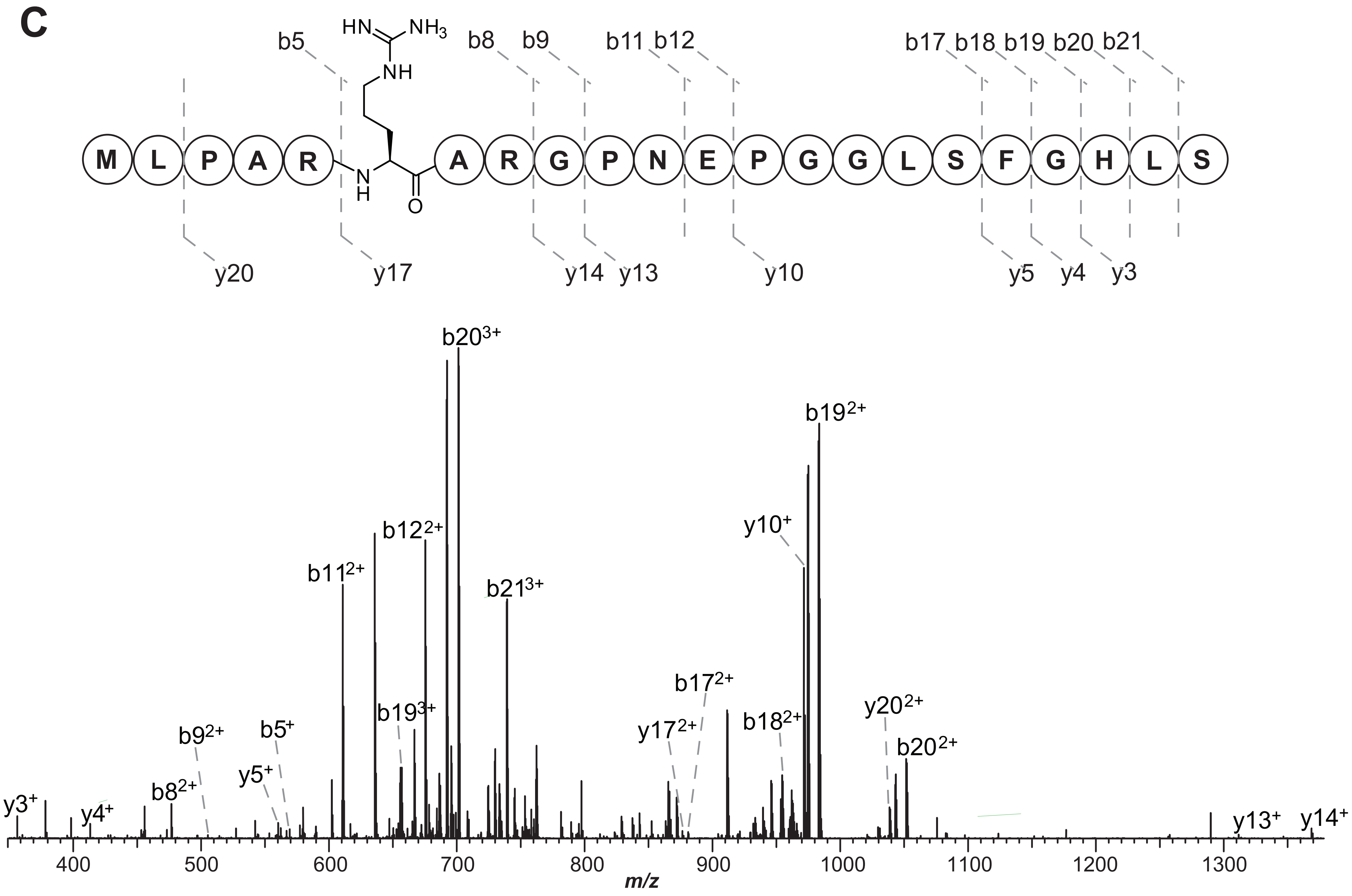

Supplement: S6 Fig — (A) The triply charged molecular ion shows the lack of a methylation (774.07 Da). (B) The 774.07-Da ion was subjected to CID with assigned ions indicated in tabular form. (C) MS/MS spectral data show no methylation on Arg285 (b8 and y17). Equivalent data were obtained with strains ΔmamAΔmcmA, ΔmamAΔycaO-tfuA, and ΔmamAΔmcmAΔycaO-tfuA. CID, collision-induced dissociation; HR-ESI MS/MS, high-resolution electrospray ionization tandem mass spectrometry; mamA, methylarginine modification; mcmA, methylcysteine modification; MS, mass spectrometry. (PDF) [file pbio.3000507.s006.pdf]
