# Supplementary material for: Functional interactions between posttranslationally modified amino acids of methyl-coenzyme M reductase in Methanosarcina acetivorans
Source: PLoS Biol. 2020 Feb 24;18(2):e3000507. doi: 10.1371/journal.pbio.3000507 (PMC7058361; doi:10.1371/journal.pbio.3000507)

A

Ion	Calculated mass (Da)	Observed mass (Da)	Error (ppm)
b5⁺	522.2711	522.2705	1.32
b6⁺	669.3396	669.3383	1.81
b7⁺	784.3665	784.3649	2.07
b8⁺	897.4506	897.4488	1.99
b9⁺	1025.5091	1025.5070	2.05
b11⁺	1266.5790	1266.5764	2.04
b12⁺	1383.6038	1383.6013	1.81
b13⁺	1440.6253	1440.6219	2.37
b14⁺	1511.6624	1511.6593	2.06
$b14^{2+}$	756.3349	756.3335	1.88
b15⁺	1612.7101	1612.7077	1.46
b16⁺	1726.7530	1726.7498	1.86
b17⁺	1825.8214	1825.8180	1.90
$b17^{2+}$	913.4144	913.4125	2.09
$b18^{2+}$	969.9564	969.9543	2.23
$b22^{2+}$	1187.5441	1187.5415	2.16
$b25^{2+}$	1338.0896	1338.0870	1.94
$b26^{2+}$	1394.6316	1394.6288	2.06
$b30^{2+}$	1621.7348	1621.7310	2.37
y3⁺	417.2457	417.2451	1.44
y4⁺	532.2726	532.2718	1.48
y5⁺	629.3254	629.3242	1.80
y6⁺	742.4094	742.4081	1.80
y7⁺	799.4309	799.4293	1.99
y8⁺	928.4735	928.4715	2.12
y9⁺	1043.5004	1043.4984	1.91
y10⁺	1100.5219	1100.5198	1.94
y11⁺	1228.5805	1228.5782	1.82
y12⁺	1391.6438	1391.6410	1.98
$y12^{2+}$	696.3256	696.3241	2.07
y13⁺	1478.6758	1478.6728	2.05
y14⁺	1591.7599	1591.7567	1.97
y15⁺	1690.8283	1690.8253	1.75
$y15^{2+}$	845.9178	845.9162	1.96
y16⁺	1804.8712	1804.8682	1.70
$y20^{2+}$	1076.0048	1076.0027	1.94
$[M+3H]^{3+}$	1139.5296	1139.5273	1.97

B

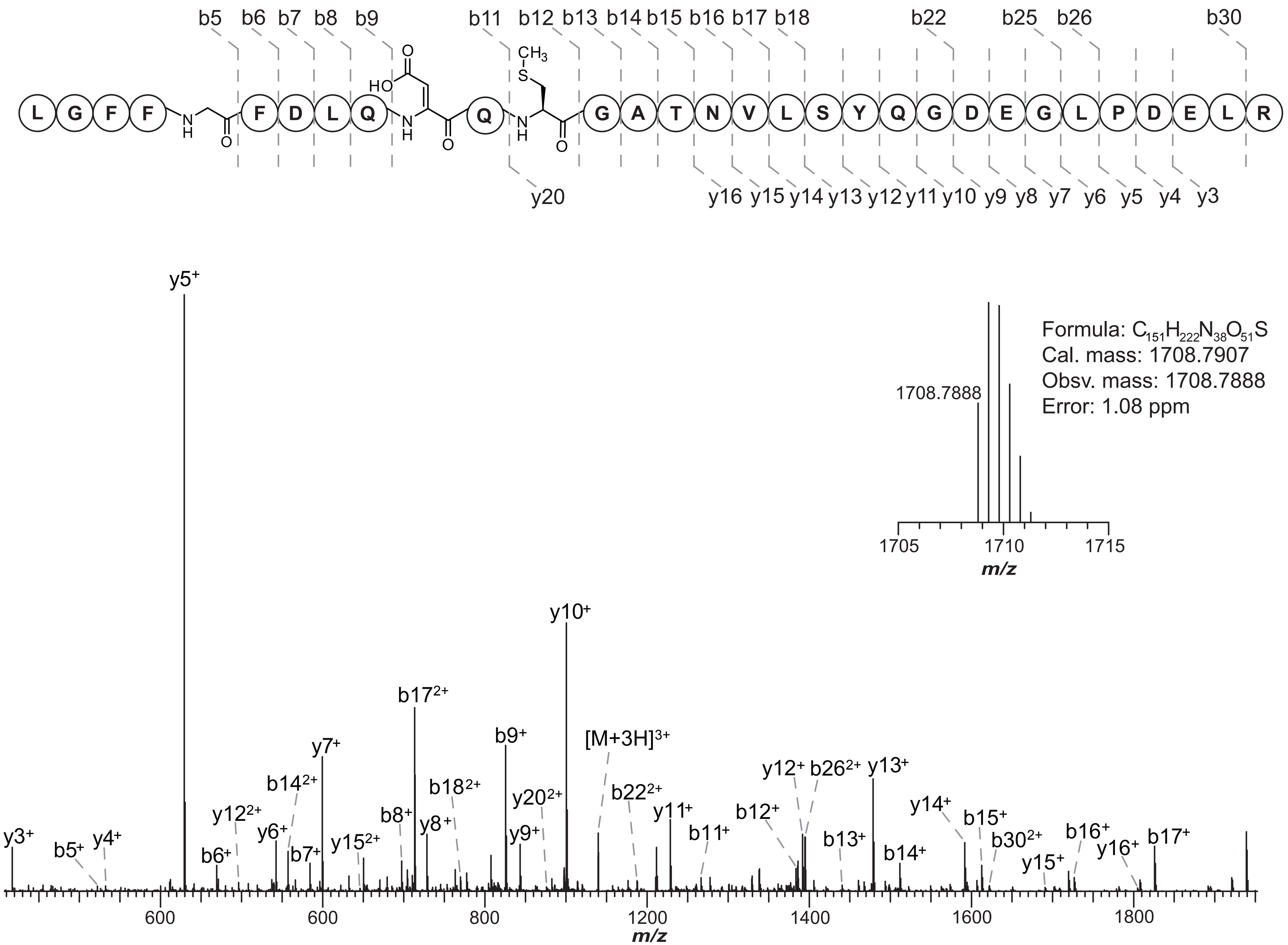

Supplement: S7 Fig — (A) The 1708.79-Da molecular ion was subjected to CID with assigned ions indicated in tabular form. (B) The doubly charged molecular ion shows the presence of a methylation and absence of thioglycine (1708.79 Da). MS/MS spectral data locate the methylation to C472 (b12 and y20) and indicate no thioglycine modification on G465 (b5). Equivalent data were obtained with strain ΔmamAΔycaO-tfuA. CID, collision-induced dissociation; HR-ESI MS/MS, high-resolutions electrospray ionization tandem mass spectrometry; mamA, methylarginine modification; MS, mass spectrometry. (PDF) [file pbio.3000507.s007.pdf]
