# Supplementary material for: Functional interactions between posttranslationally modified amino acids of methyl-coenzyme M reductase in Methanosarcina acetivorans
Source: PLoS Biol. 2020 Feb 24;18(2):e3000507. doi: 10.1371/journal.pbio.3000507 (PMC7058361; doi:10.1371/journal.pbio.3000507)

A

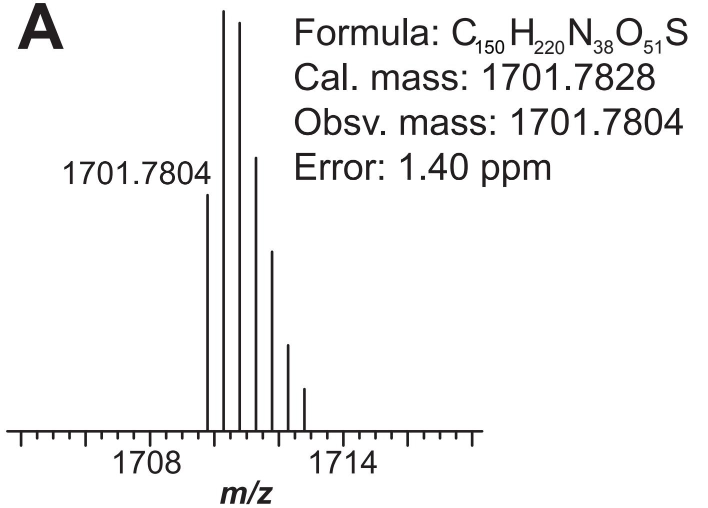

B

Ion	Calculated mass (Da)	Observed mass (Da)	Error (ppm)
$b7^{+}$	784.3665	784.3653	1.53
$b9^{+}$	1025.5091	1025.5078	1.35
$b10^{+}$	1138.5204	1138.5188	1.45
$b15^{+}$	1598.6945	1598.6927	1.07
$b17^{+}$	1811.8058	1811.8025	1.82
$b17^{2+}$	906.4066	906.4049	1.80
$b18^{+}$	1924.8899	1924.8867	1.64
$b25^{2+}$	1331.0818	1331.0795	1.71
$b26^{2+}$	1387.6238	1387.6218	1.46
$b30^{2+}$	1614.7270	1614.7242	1.73
$y5^{+}$	629.3254	629.3245	1.43
$y7^{+}$	799.4309	799.4296	1.59
$y8^{+}$	928.4735	928.4716	2.02
$y9^{+}$	1043.5004	1043.4988	1.55
$y10^{+}$	1100.5219	1100.5202	1.54
$y11^{+}$	1228.5805	1228.5787	1.46
$y12^{+}$	1391.6438	1391.6417	1.47
$y13^{+}$	1478.6758	1478.6734	1.63
$y14^{+}$	1591.7599	1591.7574	1.53
$y16^{+}$	1804.8712	1804.8672	2.21
$y17^{+}$	1905.9189	1905.9162	1.42
$[M+2H]^{2+}$	1701.7828	1701.7804	1.40

C

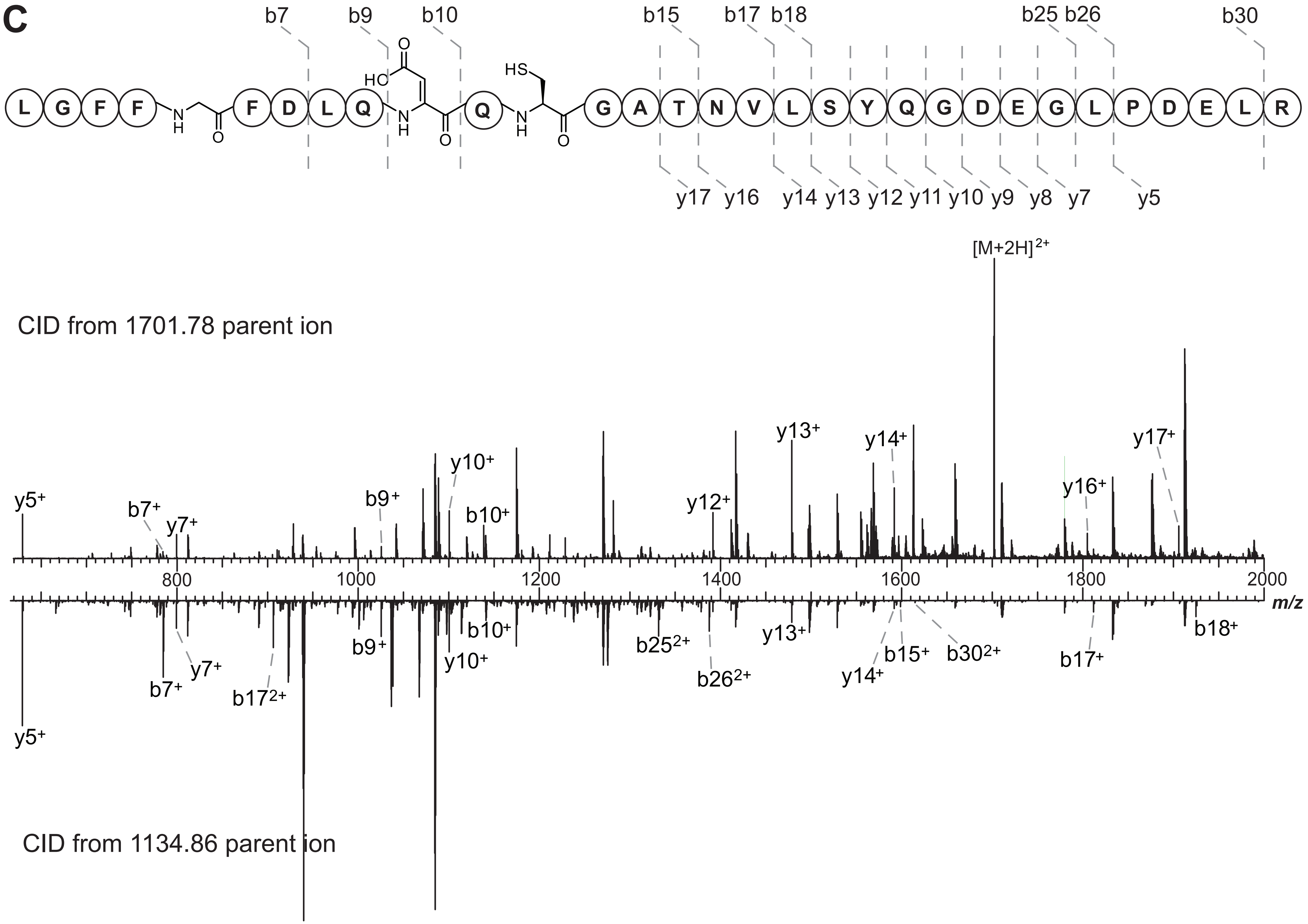

Supplement: S8 Fig — (A) The doubly charged molecular ion shows the lack of thioglycine and methylation (1701.78 Da). (B) The 1701.78-Da and 1134.86 molecular ions were subjected to CID with assigned ions indicated in tabular form. (C) MS/MS spectral data from the two parent ions indicate the lack of a thioamide (b7) and methylation on C472 (b15). Equivalent data were obtained with strain ΔmamAΔmcmAΔycaO-tfuA. CID, collision-induced dissociation; HR-ESI MS/MS, high-resolutions electrospray ionization tandem mass spectrometry; mamA, methylarginine modification; mcmA, methylcysteine modification; MS, mass spectrometry. (PDF) [file pbio.3000507.s008.pdf]
