# Supplementary material for: Functional interactions between posttranslationally modified amino acids of methyl-coenzyme M reductase in Methanosarcina acetivorans
Source: PLoS Biol. 2020 Feb 24;18(2):e3000507. doi: 10.1371/journal.pbio.3000507 (PMC7058361; doi:10.1371/journal.pbio.3000507)
Supplement: S1 Table — (DOCX) [file pbio.3000507.s010.docx]

**S1 Table:** **Data collection, and refinement statistics**

|  | **Wild-type** | **∆*ycaO-tfuA***  (thioglycine) | **∆*mamA***  (methyl-Arg) | **∆*mamA***  (methyl-Cys) | **∆*mamA/*∆*mcmA*** | **∆*mcmA/*∆*ycaO-tfuA*** | **∆*mamA/*∆*mcmA/*∆*ycaO-tfuA*** |
| --- | --- | --- | --- | --- | --- | --- | --- |
| **Data stats** |  |  |  |  |  |  |  |
| Space group | P2_1_2_1_2_1_ | P2_1_2_1_2_1_ | P2_1_ | P2_1_2_1_2_1_ | P2_1_ | C2 | P2_1_2_1_2_1_ |
| Unit cell | 88.9, 144.1, 192.2 | 85.4, 144.6, 192.8 | 108.2, 82.0, 122.8, 91.9^o^ | 80.0, 111.0, 241.0 | 107.9, 82.0, 122.7, 92.1^o^ | 166.7, 80.4, 174.4, 95.1^o^ | 266.5, 55.5, 183.7 |
| Resolution | 80-1.66 (1.67-1.66) | 80-2.05 (2.06-2.05) | 80-2.23 (2.24-2.23) | 48-2.1 (2.11-2.1) | 108-2.0 (2.01-2.0) | 87-1.99 (2.0-1.99) | 120-2.18 (2.19-2.18) |
| Total reflect. | 2,197,303 | 837,198 | 380,955 | 776,177 | 579,196 | 717,230 | 639,600 |
| Unique reflect. | 289,149 | 148,678 | 93,362 | 125,710 | 127,502 | 151,400 | 105,781 |
| R_sym_ (%)^1^ | 0.11 (0.914) | 0.10 (0.761) | 0.138 (0.670) | 0.136 (0.718) | 0.088 (0.720) | 0.146 (0.512) | 0.143 (0.552) |
| I/σ(I) ^1^ | 12.5 (2.0) | 12.3 (2.0) | 9.5 (2.0) | 11.3 (2.7) | 16.1 (1.6) | 7.6 (2.1) | 8.5 (2.1) |
| Completeness (%)^1^ | 99.4 (99.80) | 98.9 (100) | 88.6 (81.0) | 99.8 (100) | 99.0 (92.6) | 96.0 (74.8) | 95.4 (95.4) |
| Redundancy | 7.6 (7.8) | 5.6 (5.9) | 4.1 (3.5) | 6.2 (6.4) | 4.6 (3.8) | 4.7 (1.9) | 6.0 (5.1) |
| **Refinement** |  |  |  |  |  |  |  |
| Resolution (Å) | 25.0-1.66 | 25.0-2.05 | 50.0-2.23 | 25.0-2.1 | 25.0-2.0 | 25.0-2.0 | 25.0-2.18 |
| # reflections | 274,636 | 141,026 | 83,302 | 119,300 | 133,908 | 142,109 | 100,164 |
| R_work_ / R_free_^2^ | 15.5/17.8 | 15.9/19.5 | 18.8/25.4 | 14.6/19.3 | 15.9/19.8 | 16.2/20.0 | 16.0/22.2 |
| # of atoms |  |  |  |  |  |  |  |
| Protein | 18,773 | 18,766 | 18,780 | 18,770 | 18,792 | 18,778 | 18,800 |
| CoM/CoB | 14/42 | 14/42 | 14/42 | 14/42 | 14/42 | 14/42 | 14/42 |
| F_430_ | 124 | 124 | 124 | 124 | 124 | 124 | 124 |
| Water | 2788 | 1500 | 1012 | 2052 | 1,477 | 1,717 | 1,609 |
| B-factors |  |  |  |  |  |  |  |
| Protein | 17.5 | 29.1 | 26.7 | 21.1 | 25.6 | 25.4 | 27.6 |
| CoM/CoB | 13.1/13.3 | 25.7/27.6 | 22.1/20.3 | 18.8/17.2 | 19.5/25.6 | 24.9/23.9 | 25.7/24.4 |
| F_430_ | 13.2 | 22.7 | 19.9 | 16.5 | 18.6 | 19.4 | 21.5 |
| Water | 32.7 | 35.9 | 25.0 | 32.4 | 33.7 | 33.5 | 30.1 |
| Bond RMSDs |  |  |  |  |  |  |  |
| Lengths (Å) | 0.007 | 0.009 | 0.010 | 0.008 | 0.009 | 0.008 | 0.009 |
| Angles (°) | 1.288 | 1.363 | 1.52 | 1.349 | 1.361 | 1.344 | 1.438 |

1. Highest resolution shell is shown in parenthesis.

2. R-factor = Σ(|F_obs_|-k|F_calc_|)/Σ|F_obs_|and R-free is the R value for a test set of reflections consisting of a random 5% of the diffraction data not used in refinement.
