# Supplementary material for: Functional interactions between posttranslationally modified amino acids of methyl-coenzyme M reductase in Methanosarcina acetivorans
Source: PLoS Biol. 2020 Feb 24;18(2):e3000507. doi: 10.1371/journal.pbio.3000507 (PMC7058361; doi:10.1371/journal.pbio.3000507)
Supplement: S2 Table — HS, high-salt. (DOCX) [file pbio.3000507.s011.docx]

**S2 Table:** Growth rate of *Methanosarcina* strains on HS-methanol medium at 36 ^o^C.

| **Strain** | **Methanol (125 mM; 36 °C)** | | | | |
| --- | --- | --- | --- | --- | --- |
|  | **Growth Rate (GR) of 3 biological replicates (h^-1^)** | **Mean GR* (h^-1^)** | **SD GR** (h^-1^)** | **Ratio** | **p-value#** |
| WWM60 | 0.098, 0.106, 0.096 | 0.1 | 0.005 | **1** |  |
| WWM992 | 0.098, 0.101. 0.102 | 0.101 | 0.002 | **1.01** | 0.737 |
|  |  |  |  |  |  |
| WWM60 | 0.078, 0.075, 0.078 | 0.077 | 0.002 | **1** |  |
| WWM1055 | 0.071, 0.074, 0.076 | 0.074 | 0.002 | **0.096** | 0.14 |
| WWM1068 | 0.082, 0.074, 0.083 | 0.08 | 0.005 | **1.039** | 0.389 |
|  |  |  |  |  |  |
| WWM60 | 0.075, 0.076, 0.078 | 0.076 | 0.001 | **1** |  |
| WWM 1100 | 0.076, 0.075, 0.075 | 0.076 | 0.0003 | **1** | 1 |
|  |  |  |  |  |  |
| WWM60 | 0.080, 0.076, 0.083 | 0.08 | 0.004 | **1** |  |
| WWM1110 | 0.079, 0.081, 0.080 | 0.08 | 0.001 | **1** | 1 |
| WWM1107 | 0.088, 0.085, 0.088 | 0.087 | 0.003 | **1.087** | **0.042** |
|  |  |  |  |  |  |
| WWM60 | 0.078, 0.078, 0.080 | 0.079 | 0.001 | **1** |  |
| WWM1101 | 0.084, 0.084, 0.080 | 0.083 | 0.002 | **1.051** | **0.036** |
|  |  |  |  |  |  |
|  |  | * average of 3 replicates | ** standard deviation of 3 replicates |  | # unpaired t-test using averages |
