# Supplementary material for: Functional interactions between posttranslationally modified amino acids of methyl-coenzyme M reductase in Methanosarcina acetivorans
Source: PLoS Biol. 2020 Feb 24;18(2):e3000507. doi: 10.1371/journal.pbio.3000507 (PMC7058361; doi:10.1371/journal.pbio.3000507)
Supplement: S3 Table — HS, high-salt; TMA, trimethylamine. (DOCX) [file pbio.3000507.s012.docx]

**S3 Table:** Growth rate of *Methanosarcina* strains on HS-TMA medium at 36 ^o^C.

| **Strain** | **TMA (50 mM; 36 °C)** | | | | |
| --- | --- | --- | --- | --- | --- |
|  | **Growth Rate (GR) of 3 biological replicates (h^-1^)** | **Mean GR* (h^-1^)** | **SD GR** (h^-1^)** | **Ratio** | **p-value#** |
| WWM60 | 0.061, 0.067, 0.062 | 0.063 | 0.003 | **1** |  |
| WWM992 | 0.064, 0.065, 0.061 | 0.063 | 0.002 | **1** | 1 |
|  |  |  |  |  |  |
| WWM60 | 0.053, 0.054, 0.056 | 0.054 | 0.001 | **1** |  |
| WWM1055 | 0.057, 0.057, 0.058 | 0.058 | 0.0003 | **1.074** | **0.003** |
|  |  |  |  |  |  |
| WWM60 | 0.051, 0.052, 0.053 | 0.052 | 0.001 | **1** |  |
| WWM 1100 | 0.051, 0.055, 0.053 | 0.053 | 0.002 | **1.019** | 0.482 |
| WWM1101 | 0.055, 0.058, 0.054 | 0.056 | 0.002 | **1.077** | **0.036** |
|  |  |  |  |  |  |
| WWM60 | 0.055, 0.055, 0.054 | 0.055 | 0.001 | **1** |  |
| WWM1110 | 0.050, 0.049, 0.050 | 0.05 | 0.0005 | **0.909** | **0.002** |
| WWM1107 | 0.057, 0.058, 0.055 | 0.057 | 0.001 | **1.036** | 0.071 |
|  |  |  |  |  |  |
| WWM60 | 0.060, 0.060, 0.057 | 0.059 | 0.002 | **1** |  |
| WWM1068 | 0.059, 0.058, 0.054 | 0.057 | 0.002 | **0.966** | 0.288 |
|  |  |  |  |  |  |
|  |  | * average of 3 replicates | ** standard deviation of 3 replicates |  | # unpaired t-test using averages |
