# Supplementary material for: Functional interactions between posttranslationally modified amino acids of methyl-coenzyme M reductase in Methanosarcina acetivorans
Source: PLoS Biol. 2020 Feb 24;18(2):e3000507. doi: 10.1371/journal.pbio.3000507 (PMC7058361; doi:10.1371/journal.pbio.3000507)
Supplement: S4 Table — DMS, dimethyl sulfide; HS, high-salt. (DOCX) [file pbio.3000507.s013.docx]

**S4 Table:** Growth rate of *Methanosarcina* strains on HS-DMS medium at 36 ^o^C.

| **Strain** | **DMS (20 mM; 36 °C)** | | | | |
| --- | --- | --- | --- | --- | --- |
|  | **Growth Rate (GR) of 3 biological replicates (h^-1^)** | **Mean GR* (h^-1^)** | **SD GR** (h^-1^)** | **Ratio** | **p-value#** |
| WWM60 | 0.021, 0.023, 0.022 | 0.022 | 0.001 | **1** |  |
| WWM992 | 0.011, 0.012, 0.012 | 0.012 | 0.0003 | **0.054** | **<0.001** |
|  |  |  |  |  |  |
| WWM60 | 0.030, 0.032, 0.029 | 0.03 | 0.002 | 1 |  |
| WWM1055 | 0.030, 0.028, 0.029 | 0.029 | 0.001 | **0.967** | 0.482 |
| WWM1068 | 0.021, 0.019, 0.021 | 0.021 | 0.001 | **0.7** | **0.002** |
| WWM 1100 | 0.018, 0.018, 0.019 | 0.018 | 0.0002 | **0.6** | **<0.001** |
| WWM1101 | 0.019, 0.019, 0.018 | 0.019 | 0.0003 | **0.633** | **<0.001** |
| WWM1110 | No growth | 0 | 0 | **0** |  |
| WWM1107 | 0.026, 0.026, 0.026 | 0.026 | 0.0005 | **867** | **0.028** |
|  |  |  |  |  |  |
|  |  |  |  |  |  |
|  |  | * average of 3 replicates | ** standard deviation of 3 replicates |  | # unpaired t-test using averages |
