# Supplementary material for: Functional interactions between posttranslationally modified amino acids of methyl-coenzyme M reductase in Methanosarcina acetivorans
Source: PLoS Biol. 2020 Feb 24;18(2):e3000507. doi: 10.1371/journal.pbio.3000507 (PMC7058361; doi:10.1371/journal.pbio.3000507)
Supplement: S5 Table — HS, high-salt. (DOCX) [file pbio.3000507.s014.docx]

**S5 Table:** Growth rate of *Methanosarcina* strains on HS-acetate medium at 36 ^o^C.

| **Strain** | **Acetate (40 mM; 36 °C)** | | | | |
| --- | --- | --- | --- | --- | --- |
|  | **Growth Rate (GR) of 3 biological replicates (h^-1^)** | **Mean GR* (h^-1^)** | **SD GR** (h^-1^)** | **Ratio** | **p-value#** |
| WWM60 | 0.012, 0.012, 0.014 | 0.013 | 0.001 | **1** |  |
| WWM992 | 0.006, 0.006, 0.005 | 0.006 | 0.0005 | **0.461** | **<0.001** |
|  |  |  |  |  |  |
|  |  |  |  |  |  |
| WWM60 | 0.009, 0.011, 0.011 | 0.01 | 0.001 | **1** |  |
| WWM1055 | 0.008, 0.007, 0.006 | 0.007 | 0.001 | **0.7** | **0.021** |
| WWM1068 | 0.010, 0.010, 0.011 | 0.01 | 0.001 | **1** | 1 |
| WWM 1100 | 0.011, 0.011, 0.010 | 0.01 | 0.001 | **1** | 1 |
| WWM1101 | 0.009, 0.010, 0.009 | 0.009 | 0.001 | **0.9** | 0.288 |
| WWM1110 | 0.005, 0.005, 0.005 | 0.005 | 0.0003 | **0.5** | **0.001** |
| WWM1107 | 0.007, 0.008, 0.008 | 0.007 | 0.0004 | **0.7** | **0.008** |
|  |  |  |  |  |  |
|  |  | * average of 3 replicates | ** standard deviation of 3 replicates |  | # unpaired t-test using averages |
