# Supplementary material for: Functional interactions between posttranslationally modified amino acids of methyl-coenzyme M reductase in Methanosarcina acetivorans
Source: PLoS Biol. 2020 Feb 24;18(2):e3000507. doi: 10.1371/journal.pbio.3000507 (PMC7058361; doi:10.1371/journal.pbio.3000507)
Supplement: S6 Table — HS, high-salt. (DOCX) [file pbio.3000507.s015.docx]

**S6 Table:** Growth yield of *Methanosarcina* strains on HS-methanol medium at 36 ^o^C.

| **Strain** | **Methanol (125 mM; 36 °C)** | | | | |
| --- | --- | --- | --- | --- | --- |
|  | **Max OD600 of 3 biological replicates** | **Mean Yield*** | **SD Yield**** | **Ratio** | **p-value#** |
| WWM60 | 5.22, 5.66, 5.17 | 5.35 | 0.27 | **1** |  |
| WWM992 | 4.73, 5.049, 5.412 | 5.06 | 0.34 | **0.95** | 0.314 |
|  |  |  |  |  |  |
| WWM60 | 4.94, 4.18, 4.76 | 4.63 | 0.40 | **1** |  |
| WWM1055 | 3.70, 4.21, 4,67 | 4.20 | 0.49 | **0.91** | 0.297 |
| WWM1068 | 4.36, 3.88, 4.60 | 4.28 | 0.36 | **0.92** | 0.324 |
|  |  |  |  |  |  |
| WWM60 | 4.47, 3.92, 4.31 | 4.231 | 0.284 | **1** |  |
| WWM 1100 | 4.10, 3.92, 4.08 | 4.033 | 0.102 | **0.95** | 0.319 |
|  |  |  |  |  |  |
| WWM60 | 3.48, 3.23, 3.31 | 3.34 | 0.13 | **1** |  |
| WWM1110 | 3.61, 3.48, 3.36 | 3.48 | 0.13 | **1.04** | 0.234 |
| WWM1107 | 3.84, 4.00, 3.39 | 3.74 | 0.32 | **1.12** | 0.109 |
|  |  |  |  |  |  |
| WWM60 | 4.44, 3.92, 4.32 | 4.23 | 0.13 | **1** |  |
| WWM1101 | 3.82, 4.10, 4.34 | 4.09 | 0.26 | **0.97** | 0.456 |
|  |  |  |  |  |  |
|  |  | * average of 3 replicates | ** standard deviation of 3 replicates |  | # unpaired t-test using averages |
| Yield = Max. optical density at 600 nm |  |  |  |  |  |
