# Supplementary material for: Functional interactions between posttranslationally modified amino acids of methyl-coenzyme M reductase in Methanosarcina acetivorans
Source: PLoS Biol. 2020 Feb 24;18(2):e3000507. doi: 10.1371/journal.pbio.3000507 (PMC7058361; doi:10.1371/journal.pbio.3000507)
Supplement: S7 Table — HS, high-salt; TMA, trimethylamine. (DOCX) [file pbio.3000507.s016.docx]

**S7 Table:** Growth yield of *Methanosarcina* strains on HS-TMA medium at 36 ^o^C.

| **Strain** | **TMA (50 mM; 36 °C)** | | | | |
| --- | --- | --- | --- | --- | --- |
|  | **Max OD600 of 3 biological replicates** | **Mean Yield*** | **SD Yield**** | **Ratio** | **p-value#** |
| WWM60 | 9.34, 10.24, 9.13 | 9.57 | 0.59 | **1** |  |
| WWM992 | 8.88, 8.28, 9.57 | 8.91 | 0.64 | **0.93** | 0.261 |
|  |  |  |  |  |  |
| WWM60 | 4.97, 4.00, 6.06 | 5.01 | 1.03 | **1** |  |
| WWM1055 | 4.51, 4.36, 4.65 | 4.51 | 0.15 | **0.899** | 0.447 |
|  |  |  |  |  |  |
| WWM60 | 5.31, 5.71, 5.16 | 5.39 | 0.28 | **1** |  |
| WWM 1100 | 4.87, 5.09, 4.77 | 4.91 | 0.16 | **0.91** | 0.064 |
| WWM1101 | 4.71, 4.91, 4.74 | 4.78 | 0.11 | **0.89** | **0.025** |
|  |  |  |  |  |  |
| WWM60 | 8.4, 7.8, 8.04 | 8.08 | 0.30 | **1** |  |
| WWM1110 | 7.56, 7.5, 7.14 | 7.40 | 0.23 | **0.92** | **0.036** |
| WWM1107 | 8.4, 7.8, 7.92 | 8.04 | 0.32 | **0.99** | 0.882 |
|  |  |  |  |  |  |
| WWM60 | 8.83, 8.58, 8.19 | 8.54 | 0.32 | **1** |  |
| WWM1068 | 7.55, 7.81, 7.14 | 7.50 | 0.34 | **0.88** | **0.018** |
|  |  |  |  |  |  |
|  |  |  |  |  |  |
|  |  | * average of 3 replicates | ** standard deviation of 3 replicates |  | # unpaired t-test using averages |
| Yield = Max. optical density at 600 nm |  |  |  |  |  |
