# Supplementary material for: Functional interactions between posttranslationally modified amino acids of methyl-coenzyme M reductase in Methanosarcina acetivorans
Source: PLoS Biol. 2020 Feb 24;18(2):e3000507. doi: 10.1371/journal.pbio.3000507 (PMC7058361; doi:10.1371/journal.pbio.3000507)
Supplement: S8 Table — DMS, dimethyl sulfide; HS, high-salt. (DOCX) [file pbio.3000507.s017.docx]

**S8 Table:** Growth yield of *Methanosarcina* strains on HS-DMS medium at 36 ^o^C.

| **Strain** | **DMS (20 mM; 36 °C)** | | | | |
| --- | --- | --- | --- | --- | --- |
|  | **Max OD600 of 3 biological replicates** | **Mean Yield*** | **SD Yield**** | **Ratio** | **p-value#** |
| WWM60 | 0.603, 0.606, 0.596 | 0.602 | 0.005 | **1** |  |
| WWM992 | 0.455, 0.446, 0.432 | 0.444 | 0.011 | **0.737** | **<0.001** |
|  |  |  |  |  |  |
| WWM60 | 0.071, 0.645, 0.662, | 0.672 | 0.034 | 1 |  |
| WWM1055 | 0.869, 0.849, 0.787 | 0.835 | 0.043 | **1.243** | **0.007** |
| WWM1068 | 0.664, 0.707, 0.683 | 0.685 | 0.03 | **1.019** | 0.645 |
| WWM 1100 | 0.559, 0.577, 0.568 | 0.568 | 0.013 | **0.845** | **0.008** |
| WWM1101 | 0.571, 0.671, 0.571 | 0.604 | 0.058 | **0.899** | 0.155 |
| WWM1110 | No Growth | 0 | 0 | **0** |  |
| WWM1107 | 0.7, 0.666, 0.684 | 0.683 | 0.024 | **1.016** | 0.671 |
|  |  |  |  |  |  |
|  |  |  |  |  |  |
|  |  | * average of 3 replicates | ** standard deviation of 3 replicates |  | # unpaired t-test using averages |
| Yield = Max. optical density at 600 nm |  |  |  |  |  |
