# Supplementary material for: Functional interactions between posttranslationally modified amino acids of methyl-coenzyme M reductase in Methanosarcina acetivorans
Source: PLoS Biol. 2020 Feb 24;18(2):e3000507. doi: 10.1371/journal.pbio.3000507 (PMC7058361; doi:10.1371/journal.pbio.3000507)
Supplement: S9 Table — HS, high-salt. (DOCX) [file pbio.3000507.s018.docx]

**S9 Table:** Growth yield of *Methanosarcina* strains on HS-acetate medium at 36 ^o^C.

| **Strain** | **Acetate (40 mM; 36 °C)** | | | | |
| --- | --- | --- | --- | --- | --- |
|  | **Max OD600 of 3 biological replicates** | **Mean Yield*** | **SD Yield**** | **Ratio** | **p-value#** |
| WWM60 | 0.445, 0.445, 0.439 | 0.443 | 0.003 | **1** |  |
| WWM992 | 0.341, 0.36, 0.368 | 0.356 | 0.014 | **0.804** | **<0.001** |
|  |  |  |  |  |  |
|  |  |  |  |  |  |
| WWM60 | 0.33, 0.32, 0.358 | 0.336 | 0.02 | **1** |  |
| WWM1055 | 0.373, 0.291, 0.327 | 0.33 | 0.041 | **0.982** | 0.831 |
| WWM1068 | 0.353, 0.4, 0.426 | 0.403 | 0.037 | **1.199** | 0.051 |
| WWM 1100 | 0.335, 0.342, 0.36 | 0.346 | 0.013 | **1.03** | 0.051 |
| WWM1101 | 0.393, 0.394, 0.362 | 0.383 | 0.018 | **1.14** | **0.039** |
| WWM1110 | 0.262, 0.256, 0.288 | 0.269 | 0.017 | **0.801** | **0.011** |
| WWM1107 | 0.309, 0.434, 0.345 | 0.363 | 0.064 | **1.08** | 0.524 |
|  |  |  |  |  |  |
|  |  | * average of 3 replicates | ** standard deviation of 3 replicates |  | # unpaired t-test using averages |
| ** Growth yield = Max. optical density at 600 nm |  |  |  |  |  |
