# Supplementary material for: Functional interactions between posttranslationally modified amino acids of methyl-coenzyme M reductase in Methanosarcina acetivorans
Source: PLoS Biol. 2020 Feb 24;18(2):e3000507. doi: 10.1371/journal.pbio.3000507 (PMC7058361; doi:10.1371/journal.pbio.3000507)
Supplement: S10 Table — HS, high-salt; TMA, trimethylamine. (DOCX) [file pbio.3000507.s019.docx]

**S10 Table:** Growth rate of *Methanosarcina* strains on HS-TMA medium at 30 ^o^C.

| **Strain** | **TMA (50 mM; 30 °C)** | | | | |
| --- | --- | --- | --- | --- | --- |
|  | **Growth Rate (GR) of 3 biological replicates (h^-1^)** | **Mean GR* (h^-1^)** | **SD GR** (h^-1^)** | **Ratio** | **p-value#** |
| WWM60 | 0.029, 0.028, 0.028 | 0.028 | 0.0003 | **1** |  |
| WWM992 | 0.018, 0.018, 0.018 | 0.018 | 0.0003 | **0.642** | **<0.001** |
| WWM1055 | 0.033, 0.034, 0.031 | 0.033 | 0.001 | **1.179** | **<0.001** |
| WWM1068 | 0.031, 0.028, 0.032 | 0.03 | 0.002 | **1.071** | 0.162 |
| WWM 1100 | 0.035, 0.035, 0.034 | 0.035 | 0.0003 | **1.25** | **<0.001** |
| WWM1101 | 0.030, 0.033, 0.033 | 0.032 | 0.002 | **1.143** | **0.0267** |
| WWM1110 | 0.020, 0.020. 0.019 | 0.02 | 0.0005 | **0.714** | **<0.001** |
| WWM1107 | 0.034, 0.032, 0.032 | 0.033 | 0.001 | **1.179** | **0.001** |
|  |  |  |  |  |  |
|  |  | * average of 3 replicates | ** standard deviation of 3 replicates |  | # unpaired t-test using averages |
