# Supplementary material for: Functional interactions between posttranslationally modified amino acids of methyl-coenzyme M reductase in Methanosarcina acetivorans
Source: PLoS Biol. 2020 Feb 24;18(2):e3000507. doi: 10.1371/journal.pbio.3000507 (PMC7058361; doi:10.1371/journal.pbio.3000507)
Supplement: S11 Table — HS, high-salt; TMA, trimethylamine. (DOCX) [file pbio.3000507.s020.docx]

**S11 Table:** Growth rate of *Methanosarcina* strains on HS-TMA medium at 42 ^o^C.

| **Strain** | **TMA (50 mM; 42 °C)** | | | | |
| --- | --- | --- | --- | --- | --- |
|  | **Growth Rate (GR) of 3 biological replicates (h^-1^)** | **Mean GR* (h^-1^)** | **SD GR** (h^-1^)** | **Ratio** | **p-value#** |
| WWM60 | 0.024, 0.026, 0.024 | 0.025 | 0.001 | **1** |  |
| WWM992 | 0.022, 0.021, 0.021 | 0.022 | 0.001 | **0.88** | **0.021** |
| WWM1055 | 0.020, 0.019, 0.019 | 0.02 | 0.001 | **0.8** | **0.004** |
| WWM1068 | 0.032, 0.029, 0.029 | 0.03 | 0.002 | **1.2** | **0.018** |
| WWM 1100 | 0.020, 0.017, 0.016 | 0.018 | 0.002 | **0.72** | **0.006** |
| WWM1101 | 0.027, 0.024, 0.028 | 0.026 | 0.002 | **1.04** | 0.482 |
| WWM1110 | 0.021, 0.020, 0.023 | 0.021 | 0.002 | **0.84** | **0.036** |
| WWM1107 | 0.026, 0.025, 0.026 | 0.026 | 0.0005 | **1.04** | 0.196 |
|  |  |  |  |  |  |
|  |  | * average of 3 replicates | ** standard deviation of 3 replicates |  | # unpaired t-test using averages |
