# Supplementary material for: Functional interactions between posttranslationally modified amino acids of methyl-coenzyme M reductase in Methanosarcina acetivorans
Source: PLoS Biol. 2020 Feb 24;18(2):e3000507. doi: 10.1371/journal.pbio.3000507 (PMC7058361; doi:10.1371/journal.pbio.3000507)
Supplement: S12 Table — HS, high-salt; TMA, trimethylamine. (DOCX) [file pbio.3000507.s021.docx]

**S12 Table:** Growth yield of *Methanosarcina* strains on HS-TMA medium at 30 ^o^C.

| **Strain** |  | **TMA (50 mM; 30 °C)** | | | |
| --- | --- | --- | --- | --- | --- |
|  | **Max OD600 of 3 biological replicates** | **Mean Yield*** | **SD Yield**** | **Ratio** | **p-value#** |
| WWM60 | 6.03, 6.79, 5.87 | 6.23 | 0.49 | **1** |  |
| WWM992 | 3.59, 4.62, 2.66 | 3.62 | 0.98 | **0.58** | **0.014** |
| WWM1055 | 2.93, 3.16, 2.94 | 3.01 | 0.13 | **0.48** | **<0.001** |
| WWM1068 | 4.97, 4.00, 6.06 | 5.10 | 1.03 | **0.82** | 0.161 |
| WWM 1100 | 3.70, 3.49, 3.53 | 3.57 | 0.11 | **0.57** | **0.001** |
| WWM1101 | 3.00, 3.63, 3.32 | 3.32 | 0.31 | **0.53** | **0.001** |
| WWM1110 | 3.45, 3.45, 3.35 | 3.42 | 0.57 | **0.55** | **0.003** |
| WWM1107 | 3.06, 3.76, 3.10 | 3.31 | 0.39 | **0.531** | **0.001** |
|  |  |  |  |  |  |
|  |  | * average of 3 replicates | ** standard deviation of 3 replicates |  | # unpaired t-test using averages |
| ** Growth yield = Max. optical density at 600 nm |  |  |  |  |  |
