# Supplementary material for: Functional interactions between posttranslationally modified amino acids of methyl-coenzyme M reductase in Methanosarcina acetivorans
Source: PLoS Biol. 2020 Feb 24;18(2):e3000507. doi: 10.1371/journal.pbio.3000507 (PMC7058361; doi:10.1371/journal.pbio.3000507)
Supplement: S13 Table — HS, high-salt; TMA, trimethylamine. (DOCX) [file pbio.3000507.s022.docx]

**S13 Table:** Growth yield of *Methanosarcina* strains on HS-TMA medium at 42 ^o^C.

| **Strain** | **TMA (50 mM; 42 °C)** | | | | |
| --- | --- | --- | --- | --- | --- |
|  | **Max OD600 of 3 biological replicates** | **Mean Yield*** | **SD Yield**** | **Ratio** | **p-value#** |
| WWM60 | 1.320, 1.270, 1.520 | 1.37 | 0.132 | **1** |  |
| WWM992 | 0.767, 0.711, 0.720 | 0.739 | 0.04 | **0.539** | **0.001** |
| WWM1055 | 0.660,0.572, 0.831 | 0.688 | 0.132 | **0.502** | **0.003** |
| WWM1068 | 2.32, 1.780, 1.66 | 1.92 | 0.352 | **1.401** | 0.064 |
| WWM 1100 | 0.676, 0.414, 0.426 | 0.505 | 0.148 | **0.369** | **0.002** |
| WWM1101 | 2.310, 2.010, 2.090 | 2.137 | 0.155 | **1.56** | **0.003** |
| WWM1110 | 1.750, 1.890, 1.950 | 1.863 | 0.103 | **1.36** | **0.007** |
| WWM1107 | 1.690, 1.450, 1.760 | 1.633 | 0.163 | **1.192** | 0.096 |
|  |  |  |  |  |  |
| *unpaired t-test w/ WT |  |  |  |  |  |
| ** Growth yield = Max. optical density at 600 nm |  |  |  |  |  |
