# Supplementary material for: Functional interactions between posttranslationally modified amino acids of methyl-coenzyme M reductase in Methanosarcina acetivorans
Source: PLoS Biol. 2020 Feb 24;18(2):e3000507. doi: 10.1371/journal.pbio.3000507 (PMC7058361; doi:10.1371/journal.pbio.3000507)
Supplement: S14 Table — MCR, methyl-coenzyme M reductase. (DOCX) [file pbio.3000507.s023.docx]

**S14 Table:** Melting temperature of MCR complex derived from *M. acetivorans* strains as indicated.

| **Sample** | **Replicate 1 (**°C) | **Replicate 2 (**°C) | **Replicate 3 (**°C) | **Average Tm (**°C) | **Standard Deviation Tm (**°C) |
| --- | --- | --- | --- | --- | --- |
| WT | 59.5 | 60.6 | 59.5 | 59.5 | 60.6 |
| ∆*mamA* | 62.6 | 62.3 | 62.3 | 62.4 | 0.2 |
| ∆*mcmA* | 53.4 | 53.3 | 52.6 | 53.1 | 0.4 |
| ∆*ycaO-tfuA* | 60.6 | 60.3 | 59.9 | 60.3 | 0.3 |
|  |  |  |  |  |  |
| WT | 58.6 | 58.6 | 58.6 | 58.6 | 0 |
| ∆*mamA*∆*mcmA* | 51 | 51 | 51.4 | 51.1 | 0.2 |
| ∆*mamA*∆*ycaO-tfuA* | 59.6 | 59.6 | 59.6 | 59.6 | 0 |
| ∆*mcmA*∆*ycaO-tfuA* | 61.2 | 61.7 | 61.7 | 61.5 | 0.3 |
|  |  |  |  |  |  |
| WT | 59.6 | 59.6 | 59.6 | 59.6 | 0 |
| ∆*ycaO-tfuA*∆*mamA*∆*mcmA* | 60.6 | 60.6 | 60.6 | 60.6 | 0 |
|  |  |  |  |  |  |
