# Supplementary material for: Functional interactions between posttranslationally modified amino acids of methyl-coenzyme M reductase in Methanosarcina acetivorans
Source: PLoS Biol. 2020 Feb 24;18(2):e3000507. doi: 10.1371/journal.pbio.3000507 (PMC7058361; doi:10.1371/journal.pbio.3000507)
Supplement: S15 Table — (DOCX) [file pbio.3000507.s024.docx]

**S15 Table:** List of primers used in this study

| Primer | Sequence  (underlined red sequence represents overhangs for Gibson assembly) |
| --- | --- |
| *mam*_us_f | **CCTTTTGGAGCCTTTTTTTTTCGAAGTTTAAAC**GAT TGA GGA GAG AGC TGC TG |
| *mam* _us_r | TCC GCC TAC GTC GAC AAC TAC |
| *mam* _ds_f | **AATATGGAAGTAGTTGTCGACGTAGGCGGA**GGC ATG ACA AGG GAA GAA GTT C |
| *mam* _ds_r | **GATGTTGTTGGCGCGCCTGCAGGTTTAAAC**AAA GAT GGA TGC TCC TCT GG |
| *mcm*_us_f | **CCTTTTGGAGCCTTTTTTTTTCGAAGTTTAAAC**CTG ATT AGG TGC AGA TTG GTG |
| *mcm*_us_r | GCA ATC GTT GCA TGG TTT ATA G |
| *mcm*_ds_f | **GAATTATTCTATAAACCATGCAACGATTGC**GCA GGC AAA TTA GGC AGA AAA G |
| *mcm*_ds_r | **GATGTTGTTGGCGCGCCTGCAGGTTTAAAC**TCC TCG ACA ACA ACA CCT AC |
| *mam*_del_ver_f | ATA GAC TCG ATA ACG GTT CC |
| *mam*_del_ver_r | CTG CAT AAT GCT CCT TAT GG |
| *mcm*_del_ver_f | TCA CCG GAG GAG AAC TGA AAG |
| *mcm*_del_ver_r | AGA TTG CCC TTG AAG TAG TC |
| *mcrG_*N-TAP_ver_f | TAC CCA TTC AAT GAC TTC TGC |
| *mcrG_*N-TAP_ver_r | GCAC AGA TTG AAA TGC ACA AG |
