# Supplementary material for: Functional interactions between posttranslationally modified amino acids of methyl-coenzyme M reductase in Methanosarcina acetivorans
Source: PLoS Biol. 2020 Feb 24;18(2):e3000507. doi: 10.1371/journal.pbio.3000507 (PMC7058361; doi:10.1371/journal.pbio.3000507)
Supplement: S16 Table — (DOCX) [file pbio.3000507.s025.docx]

**S16 Table:** List of plasmids used in this study

| Plasmid | Features | Source |
| --- | --- | --- |
| pAMG40 | Vector for fosmid retrofitting that contains pC2A and λattB | (Guss et al. 2008) |
| pJK027A | Vector with P*mcrB*(*tetO1*) promoter fusion to *uidA* that contains φC31-attB and λattP | (Guss et al. 2008) |
| pDN201 | pJK027A-derived plasmid with P*mcrB*(*tetO1*) promoter fusion to Spy *cas9* | (Nayak and Metcalf 2017) |
| pDN247 | Cas9-based gene editing plasmid derived from pDN201 expressing two sgRNAs targeting the *ycaO-tfuA* locus and a homology repair template with 1 kb upstream and downstream flanks to generate an in-frame deletion of the *ycaO-tfuA* locus in *Methanosarcina acetivorans* | (Nayak et al 2018) |
| pDN310 | pDN201-derived plasmid with a synthetic fragment containing P*mtaCB1* promoter fusion to two sgRNAs in tandem targeting the MA4551(*mam*) locus in *M. acetivorans* | This study |
| pDN311 | pDN310-derived plasmid with a homology template comprising of 1 kb upstream and downstream flanks to generate an in-frame deletion of the MA4551 (*mam)* locus in *M. acetivorans* | This study |
| pDN312 | Cointegrate of pDN310 and pAMG40 | This study |
| pDN313 | Cointegrate of pDN311 and pAMG40 | This study |
| pDN322 | pDN201-derived plasmid with a synthetic fragment containing P*mtaCB1* promoter fusion to two sgRNAs in tandem targeting the MA4545 (*mcm*) locus in *M. acetivorans* | This study |
| pDN323 | pDN322-derived plasmid with a homology template comprising of 1 kb upstream and downstream flanks to generate an in-frame deletion of the MA4545 (*mcm)* locus in *M. acetivorans* | This study |
| pDN324 | Cointegrate of pDN322 and pAMG40 | This study |
| pDN325 | Cointegrate of pDN323 and pAMG40 | This study |
| pDN303 | pDN201-derived plasmid with a synthetic DNA fragment containing P*mtaCB1* promoter fusion to a sgRNA targeting the N-terminus of the *mcrG* locus in *M. acetivorans* | Nayak et al 2018 |
| pDN327 | pDN303-derived plasmid with a homology repair template to introduce an Enterokinase cleavable tandem affinity purification (TAP) tag comprising of a 3X FLAG tag and a Twin-Strep tag | This study |
| pDN329 | Cointegrate of pDN327 and pAMG40 | This study |

**References**

1. Guss AM, Rother M, Zhang JK, Kulkarni G, Metcalf WW (2008) New Methods for Tightly Regulated Gene Expression and Highly Efficient Chromosomal Integration of Cloned Genes for *Methanosarcina* Species. *Archaea* 2(3):193–203.
2. Nayak DD, Metcalf WW (2017) Cas9-mediated Genome Editing in the Methanogenic Archaeon *Methanosarcina acetivorans*. *Proc Natl Acad Sci U S A* 114(11):2976-2981.
3. Nayak DD, Mahanta N, Mitchell DA, Metcalf WW (2017) Post-translational Thioamidation of Methyl-Coenzyme M Reductase, a Key Enzyme in Methanogenic and Methanotrophic archaea. *Elife* 6(I):1–18.
