# Supplementary material for: Functional interactions between posttranslationally modified amino acids of methyl-coenzyme M reductase in Methanosarcina acetivorans
Source: PLoS Biol. 2020 Feb 24;18(2):e3000507. doi: 10.1371/journal.pbio.3000507 (PMC7058361; doi:10.1371/journal.pbio.3000507)
Supplement: S17 Table — (DOCX) [file pbio.3000507.s026.docx]

**S17 Table:** List of target sequences used in this study

| Locus tag  (gene) | Target sequence (+ PAM) | Position on *M. acetivorans* chromosome |
| --- | --- | --- |
| MA4551 (*mam*) | AAACCTTTGCAGTCTACCCC **GGG** | 5602064-5602084 (- strand) |
| MA4551 (*mam*) | GTCTACCCCGTCTCTTCTAA **GGG** | 5603100-5603122 (- strand) |
| MA4545  (*mcm*) | AGGTTAATCTGTTCTGCCAG **AGG** | 5595565-5595587 (- strand) |
| MA4545  (*mcm*) | AATTCTGCTGTATCCACACC **GGG** | 5596354-5596376 (- strand) |
| MA4547  (*mcrG*) | CTTTCTTCTGTTAGCGCCGA **CGG** | 5599085-5599107 (- strand) |
