# Supplementary material for: Functional interactions between posttranslationally modified amino acids of methyl-coenzyme M reductase in Methanosarcina acetivorans
Source: PLoS Biol. 2020 Feb 24;18(2):e3000507. doi: 10.1371/journal.pbio.3000507 (PMC7058361; doi:10.1371/journal.pbio.3000507)
Supplement: S18 Table — (DOCX) [file pbio.3000507.s027.docx]

**S18 Table:** List of *Methanosarcina acetivorans* strains used in this study

| Strain | Genotype | Construction details | Source |
| --- | --- | --- | --- |
| WWM60 | Δ*hpt*::*PmcrB-tetR* | --- | (Guss et al. 2008) |
| WWM992 | Δ*hpt*::*PmcrB-tetR,*  Δ*ycaO-tfuA* | WWM60 was transformed to Pur^R^ with pDN247; plasmid-cured strain was isolated by plating on medium with 8ADP | (Nayak et al 2018) |
| WWM1055 | Δ*hpt*::*PmcrB-tetR,* ΔMA4551 (∆*mam)* | WWM60 was transformed to Pur^R^ with pDN313; plasmid-cured strain was isolated by plating on medium with 8ADP | This study |
| WWM1068 | Δ*hpt*::*PmcrB-tetR,* ΔMA4545 *(∆mcm)* | WWM60 was transformed to Pur^R^ with pDN325; plasmid-cured strain was isolated by plating on medium with 8ADP | This study |
| WWM1086 | Δ*hpt*::*PmcrB-tetR,* Enterokinase cleavable TAP-tag at N-terminus of *mcrG* | WWM60 was transformed to Pur^R^ with pDN329; plasmid-cured strain was isolated by plating on medium with 8ADP | This study |
| WWM1100 | Δ*hpt*::*PmcrB-tetR,* Δ*mam*, Δ*ycaO-tfuA* | WWM1055 was transformed to Pur^R^ with pDN247; plasmid-cured strain was isolated by plating on medium with 8ADP | This study |
| WWM1101 | Δ*hpt*::*PmcrB-tetR,* Δ*mam*, Δ*mcm* | WWM1055 was transformed to Pur^R^ with pDN325; plasmid-cured strain was isolated by plating on medium with 8ADP | This study |
| WWM1107 | Δ*hpt*::*PmcrB-tetR,* Δ*mam*, Δ*ycaO-tfuA*, Δ*mcm* | WWM1100 was transformed to Pur^R^ with pDN325; plasmid-cured strain was isolated by plating on medium with 8ADP | This study |
| WWM1110 | Δ*hpt*::*PmcrB-tetR,*  Δ*ycaO-tfuA*, Δ*mcm* | WWM992 was transformed to Pur^R^ with pDN325; plasmid-cured strain was isolated by plating on medium with 8ADP | This study |
| WWM1125 | Δ*hpt*::*PmcrB-tetR, Δ*MA*mam*, Enterokinase cleavable TAP-tag at N-terminus of *mcrG* | WWM1055 was transformed to Pur^R^ with pDN329; plasmid-cured strain was isolated by plating on medium with 8ADP | This study |
| WWM1126 | Δ*hpt*::*PmcrB-tetR, Δmcm*, Enterokinase cleavable TAP-tag at N-terminus of *mcrG* | WWM1068 was transformed to Pur^R^ with pDN329; plasmid-cured strain was isolated by plating on medium with 8ADP | This study |
| WWM1127 | Δ*hpt*::*PmcrB-tetR,* Δ*mam*, Δ*mcm*, Enterokinase cleavable TAP-tag at N-terminus of *mcrG* | WWM1101 was transformed to Pur^R^ with pDN329; plasmid-cured strain was isolated by plating on medium with 8ADP | This study |
| WWM1128 | Δ*hpt*::*PmcrB-tetR,* Δ*mam*, Δ*ycaO-tfuA,* Enterokinase cleavable TAP-tag at N-terminus of *mcrG* | WWM1100 was transformed to Pur^R^ with pDN329; plasmid-cured strain was isolated by plating on medium with 8ADP | This study |
| WWM1129 | Δ*hpt*::*PmcrB-tetR,* Δ*mam*, Δ*ycaO-tfuA*, Δ*mcm*, Enterokinase cleavable TAP-tag at N-terminus of *mcrG* | WWM1107 was transformed to Pur^R^ with pDN329; plasmid-cured strain was isolated by plating on medium with 8ADP | This study |
| WWM1134 | Δ*hpt*::*PmcrB-tetR,* Enterokinase cleavable TAP-tag at N-terminus of *mcrG*, Δ*ycaO-tfuA* | WWM1086 was transformed to Pur^R^ with pDN247; plasmid-cured strain was isolated by plating on medium with 8ADP | This study |
| WWM1135 | Δ*hpt*::*PmcrB-tetR, Δmcm*, Enterokinase cleavable TAP-tag at N-terminus of *mcrG*, Δ*ycaO-tfuA* | WWM1126 was transformed to Pur^R^ with pDN247; plasmid-cured strain was isolated by plating on medium with 8ADP | This study |

**References**

1. Guss AM, Rother M, Zhang JK, Kulkarni G, Metcalf WW (2008) New Methods for Tightly Regulated Gene Expression and Highly Efficient Chromosomal Integration of Cloned Genes for *Methanosarcina* Species. *Archaea* 2(3):193–203.
2. Nayak DD, Mahanta N, Mitchell DA, Metcalf WW (2017) Post-translational Thioamidation of Methyl-Coenzyme M Reductase, a Key Enzyme in Methanogenic and Methanotrophic archaea. *Elife* 6(I):1–18.
