# Supplementary material for: Functional interactions between posttranslationally modified amino acids of methyl-coenzyme M reductase in Methanosarcina acetivorans
Source: PLoS Biol. 2020 Feb 24;18(2):e3000507. doi: 10.1371/journal.pbio.3000507 (PMC7058361; doi:10.1371/journal.pbio.3000507)
Supplement: S1 Text — (DOCX) [file pbio.3000507.s028.docx]

**Supplementary Figure S1: HR-ESI MS/MS analysis of an MCR tryptic peptide (L_461_-R_491_, m/z 3418 Da) from the *mcmA* mutant. Panel A)** The 1140.18 Da molecular ion was subjected to CID with assigned ions indicated in tabular form. **Panel B)** The triply charged molecular ion shows the presence of a thioamide and absence of the methylcysteine (1140.18 Da). MS/MS spectral data indicates that the thioamide is located at the Gly465 (b4 and b6) and there is no methylation on Cys472 (b12 and y23). Equivalent data were obtained with strain Δ*mam*Δ*mcm*.
