# Supplementary material for: Functional interactions between posttranslationally modified amino acids of methyl-coenzyme M reductase in Methanosarcina acetivorans
Source: PLoS Biol. 2020 Feb 24;18(2):e3000507. doi: 10.1371/journal.pbio.3000507 (PMC7058361; doi:10.1371/journal.pbio.3000507)
Supplement: S2 Text — (DOCX) [file pbio.3000507.s029.docx]

**Supplementary Figure S2: MALDI-TOF MS analysis of McrA. Panel A)** Spectrum obtained from trypsinolysis of MCR obtained from WT (WWM60) and mutants lacking *ycaO-tfuA, mcmA,* and *mamA* in all possible combinations. The H_271_-R_284_ peptide contains His271 (red) that is modified to 3-methyhistidine*.* **Panel B)** Spectrum obtained from trypsinolysis of MCR from strains mentioned above. The F_408_-R_421_ peptide contains Gln420 (red) that is unmodified in *M. acetivorans*. Individual spectra are labeled with numbers in parentheses as indicated in Figure 3A.
