# Supplementary material for: Functional interactions between posttranslationally modified amino acids of methyl-coenzyme M reductase in Methanosarcina acetivorans
Source: PLoS Biol. 2020 Feb 24;18(2):e3000507. doi: 10.1371/journal.pbio.3000507 (PMC7058361; doi:10.1371/journal.pbio.3000507)
Supplement: S3 Text — (DOCX) [file pbio.3000507.s030.docx]

**Supplementary Figure S3: Panel A)** A schematic outlining the order in which *ycaO-tfuA, mamA,* and *mcmA* were deleted to generate single -double- and triple-deletion mutants using the corresponding Cas9-based gene editing vectors (pDN247 for ∆*ycaO-tfuA* in green; pDN313 for ∆*mamA* in blue; pDN325 for ∆*mcmA* in red). **Panel B)** A schematic outlining the order in which a N-terminal tandem-affinity purification (TAP) tag was introduced at the *mcrG* locus in WWM60 (WT) as well as strains lacking *ycaO-tfuA, mamA,* and *mcmA* in all possible combinations using the Cas9-based gene editing vector pDN329 (in red). We were unable to introduce the TAP-tag at the N-terminus of *mcrG* in WWM992 or WW1110 using pDN329; however, it was possible to delete the *ycaO-tfuA* locus in WWM1086 and WWM1126 using the Cas9-based gene editing vectors pDN247 (in green).
