# Supplementary material for: Functional interactions between posttranslationally modified amino acids of methyl-coenzyme M reductase in Methanosarcina acetivorans
Source: PLoS Biol. 2020 Feb 24;18(2):e3000507. doi: 10.1371/journal.pbio.3000507 (PMC7058361; doi:10.1371/journal.pbio.3000507)
Supplement: S4 Text — (DOCX) [file pbio.3000507.s031.docx]

**Supplementary Figure S4:** A maximum-likelihood phylogenetic tree of the amino acid sequence of *mamA* homologs in archaea. The node labels indicate support values calculated using the Shiomdaira-Hasegawa test using 1,000 resamples. Support values less than 0.6 have not been shown. The outgroup derives from bacterial MamA homologs (in black).
