# Supplementary material for: Functional interactions between posttranslationally modified amino acids of methyl-coenzyme M reductase in Methanosarcina acetivorans
Source: PLoS Biol. 2020 Feb 24;18(2):e3000507. doi: 10.1371/journal.pbio.3000507 (PMC7058361; doi:10.1371/journal.pbio.3000507)
Supplement: S5 Text — (DOCX) [file pbio.3000507.s032.docx]

**Supplementary Figure S5: HR-ESI MS/MS analysis of an AspN-GluC double digest peptide from wild-type MCR (M_280_-S_301_, m/z 2334 Da). Panel A)** The 4^+^ molecular ion shows the presence of a methylation (584.31 Da). **Panel B)** The 584.31 Da ion was subjected to CID with assigned ions indicated in tabular form. **Panel C)** MS/MS spectral data locates the methylation to Arg285 (b6 and y17). Equivalent data were obtained with strains Δ*ycaO-tfuA,* Δ*mcm,* and Δ*mcm*Δ*ycaO-tfuA*.
