# Supplementary material for: Functional interactions between posttranslationally modified amino acids of methyl-coenzyme M reductase in Methanosarcina acetivorans
Source: PLoS Biol. 2020 Feb 24;18(2):e3000507. doi: 10.1371/journal.pbio.3000507 (PMC7058361; doi:10.1371/journal.pbio.3000507)
Supplement: S6 Text — (DOCX) [file pbio.3000507.s033.docx]

**Supplementary Figure S6: High-resolutions electrospray ionization tandem mass spectrometry (HR-ESI MS/MS) analysis of an AspN-GluC double digest peptide from the *mamA* mutant (M_280_-S_301_, m/z 2320 Da). Panel A)** The triply charged molecular ion shows the lack of a methylation (774.07 Da). **Panel B)** The 774.07 Da ion was subjected to CID with assigned ions indicated in tabular form. **Panel C)** MS/MS spectral data shows no methylation on Arg285 (b8 and y17). Equivalent data were obtained with strains Δ*mam*Δ*mcm,* Δ*mam*Δ*ycaO-tfuA,* and Δ*mam*Δ*mcm*Δ*ycaO-tfuA*.
