# Supplementary material for: Functional interactions between posttranslationally modified amino acids of methyl-coenzyme M reductase in Methanosarcina acetivorans
Source: PLoS Biol. 2020 Feb 24;18(2):e3000507. doi: 10.1371/journal.pbio.3000507 (PMC7058361; doi:10.1371/journal.pbio.3000507)
Supplement: S7 Text — (DOCX) [file pbio.3000507.s034.docx]

**Supplementary Figure S7: HR-ESI MS/MS analysis of a tryptic peptide from the *ycaO-tfuA* mutant (L_461_-R_491_, m/z 3416 Da). Panel A)** The 1708.79 Da molecular ion was subjected to CID with assigned ions indicated in tabular form. **Panel B)** The doubly charged molecular ion shows the presence of a methylation and absence of thioglycine (1708.79 Da). MS/MS spectral data locates the methylation to C^472^ (b12 and y20) and indicates no thioglycine modification on G^465^ (b5). Equivalent data were obtained with strain Δ*mam*Δ*ycaO-tfuA*.
