# Supplementary material for: Functional interactions between posttranslationally modified amino acids of methyl-coenzyme M reductase in Methanosarcina acetivorans
Source: PLoS Biol. 2020 Feb 24;18(2):e3000507. doi: 10.1371/journal.pbio.3000507 (PMC7058361; doi:10.1371/journal.pbio.3000507)
Supplement: S8 Text — (DOCX) [file pbio.3000507.s035.docx]

**Supplementary Figure S8: HR-ESI MS/MS analysis of a tryptic peptide from the the *ycaO-tfuA*, *mcmA* double mutant (L_461_-R_491_, m/z 3402 Da). Panel A)** The doubly charged molecular ion shows the lack of thioglycine and methylation (1701.78 Da). **Panel B)** The 1701.78 Da and 1134.86 molecular ions were subjected to CID with assigned ions indicated in tabular form. **Panel C)** MS/MS spectral data from the two parent ions indicates the lack of a thioamide (b7) and methylation on C^472^ (b15). Equivalent data were obtained with strain Δ*mam*Δ*mcm*Δ*ycaO-tfuA*.
