# Supplementary material for: Functional interactions between posttranslationally modified amino acids of methyl-coenzyme M reductase in Methanosarcina acetivorans
Source: PLoS Biol. 2020 Feb 24;18(2):e3000507. doi: 10.1371/journal.pbio.3000507 (PMC7058361; doi:10.1371/journal.pbio.3000507)
Supplement: S9 Text — (DOCX) [file pbio.3000507.s036.docx]

**Supplementary Figure S9:** An overview of the methylotrophic and aceticlastic methanogenic pathways in *Methanosarcina acetivorans.* Methyl-transfer reactions from methylotrophic substrates like methanol (CH_3_OH) trimethylamine (TMA, (CH_3_)_3_NH_3_^+^) dimethylsulfide (DMS, CH_3_-S-CH_3_) lead to the generation of methyl-coenzyme M (CH_3_-CoM) which is disproportionated to methane (CH_4_) and carbon dioxide (CO_2_; metabolic flux is shown as orange arrows). Notably, the first step in oxidation of CH_3_-CoM to CO_2_ is the energy-requiring transfer of the methyl moiety to generate methyl-tetrahydrosarcinapterin (CH_3_-H_4_SPt). In contrast, aceticlastic methanogenesis leads to the formation of CH_3_-H_4_SPt, followed by reduction to CH_4_ (red arrows). Thus, the second step of the pathway is exergonic.
