# Supplementary material for: Functional interactions between posttranslationally modified amino acids of methyl-coenzyme M reductase in Methanosarcina acetivorans
Source: PLoS Biol. 2020 Feb 24;18(2):e3000507. doi: 10.1371/journal.pbio.3000507 (PMC7058361; doi:10.1371/journal.pbio.3000507)
Supplement: S4 Fig — The node labels indicate support values calculated using the Shiomdaira–Hasegawa test using 1,000 resamples. Support values less than 0.6 have not been shown. The outgroup derives from bacterial MamA homologs (in black). mamA, methylarginine modification. (PDF) [file pbio.3000507.s004.pdf]

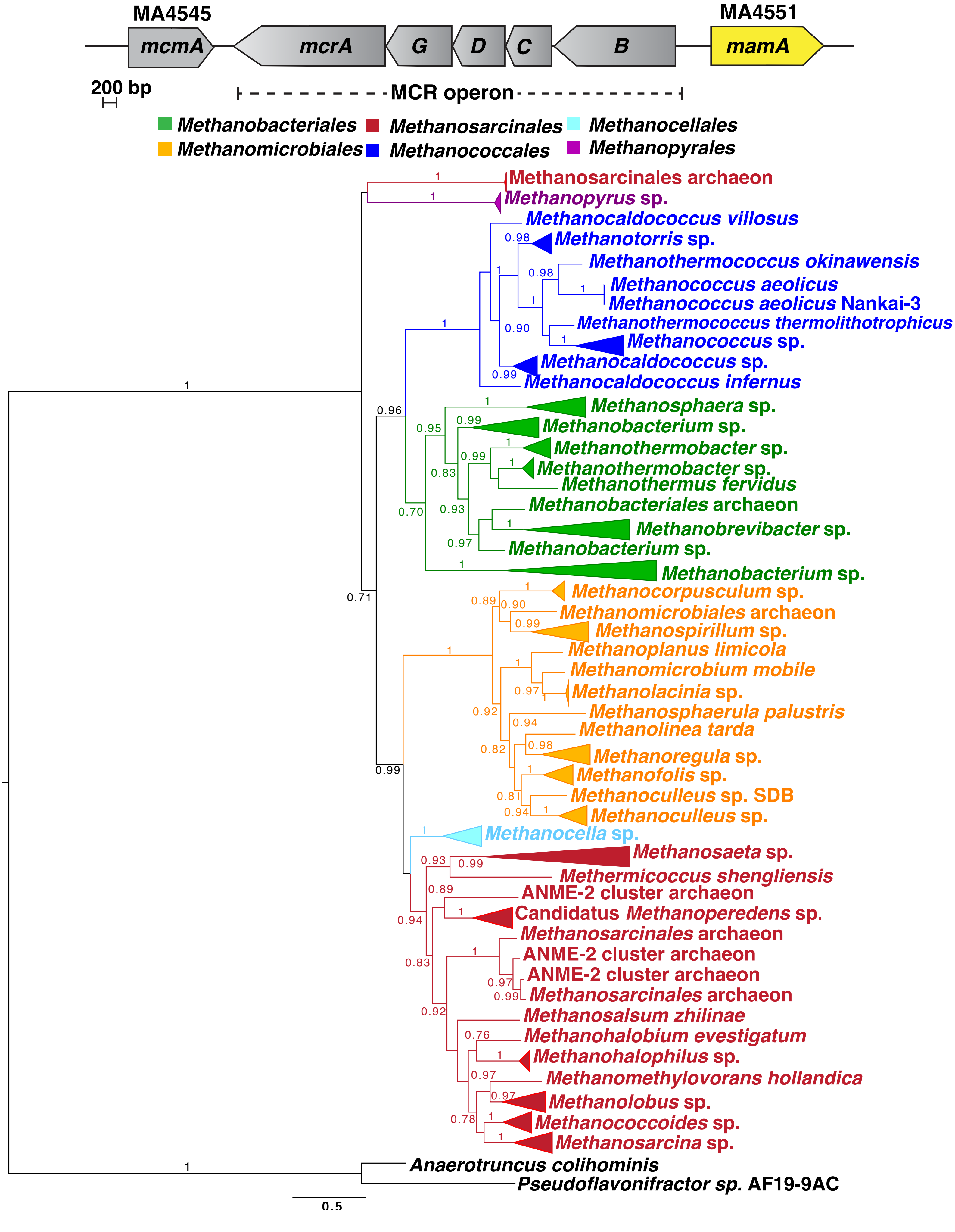

MA4545
MA4551
mcmA
mcrA
G
D
C
B
mamA
200 bp
MCR operon
Methanobacteriales
Methanosarcinales
Methanocellales
Methanomicrobiales
Methanococcales
Methanopyrales
Methanosarcinales archaeon
Methanopyrus sp.
Methanocaldococcus villosus
Methanotorris sp.
Methanothermococcus okinawensis
Methanococcus aeolicus
Methanococcus aeolicus Nankai-3
Methanothermococcus thermolithotrophicus
Methanococcus sp.
Methanocaldococcus sp.
Methanocaldococcus infernus
Methanosphaera sp.
Methanobacterium sp.
Methanothermobacter sp.
Methanothermobacter sp.
Methanothermus fervidus
Methanobacteriales archaeon
Methanobrevibacter sp.
Methanobacterium sp.
Methanobacterium sp.
Methanocorpusculum sp.
Methanomicrobiales archaeon
Methanospirillum sp.
Methanoplanus limicola
Methanomicrobium mobile
Methanolacinia sp.
Methanosphaerula palustris
Methanolinea tarda
Methanoregula sp.
Methanofolis sp.
Methanoculleus sp. SDB
Methanoculleus sp.
Methanocella sp.
Methanosaeta sp.
Methermicoccus shengliensis
ANME-2 cluster archaeon
Candidatus Methanoperedens sp.
Methanosarcinales archaeon
ANME-2 cluster archaeon
ANME-2 cluster archaeon
Methanosarcinales archaeon
Methanosalsum zhilinae
Methanohalobium evestigatum
Methanohalophilus sp.
Methanomethylovorans hollandica
Methanolobus sp.
Methanococcoides sp.
Methanosarcina sp.
Anaerotruncus colihominis
Pseudoflavonifractor sp. AF19-9AC
0.5
