# Supplementary material for: Functional interactions between posttranslationally modified amino acids of methyl-coenzyme M reductase in Methanosarcina acetivorans
Source: PLoS Biol. 2020 Feb 24;18(2):e3000507. doi: 10.1371/journal.pbio.3000507 (PMC7058361; doi:10.1371/journal.pbio.3000507)
Supplement: Attachment — Submitted filename: Nayak_et_al_point_by_point_response_121519.docx [file pbio.3000507.s037.docx]

**Point-by-point response to the reviews of PBIOLOGY-D-19-02599R1**

We thank the reviewers for their time and constructive comments, which we have addressed as indicated below. We have copied the reviewer’s comments verbatim. Our response follows each item in **red** text.

Reviewer #1: Prof. Dr. Rudolf K. Thauer, signed review

Very good work. Only two comments:

(i) In the last sentence in the abstract and the respective result section it should be stated that the structures obtained were those of inactive MCR in the Ni(II) oxidation state. The structure of active MCR in the Ni(I) oxidation state is not known. In the absence of coenzyme B the active Ni(I) state exhibits an axial N(I) based EPR signal, which is converted into a rhombic Ni(I) based EPR signal indicating major conformational changes upon coenzymes binding.

We have modified the abstract (see lines 38-39) and the results section (see lines 280) to indicate that the structures of MCR obtained in this study contain Ni in the +2 oxidation state.

(ii) It should be discussed that the post-translational modifications of McrA observed are unique for methyl-coenzyme M reductase. Other enzymes isolated and characterized from methanogenic archaea do not show any post-translational modifications: e. g. F420-reducing hydrogenases, formyl-methanofuran dehydrogenase and the HdrABC-MvhADG complex. Thus the McrA post-translational modifications must have a function unique for this enzyme.

We have also modified the discussion section to specify that the modifications found in McrA are unique to this enzyme and therefore must have functions specific to this enzyme (see lines 300-301).

---------------


Reviewer #2:
Overall: • The authors have have provided important information on the role of three post-translationally modified amino acids near the active site of methyl-coenzyme M reductase (MCR), which plays an important role in mediating global levels of methane. Specifically, they studied the thioGly465 residue (using ΔycaO-tfuA), methyl-Cys472 (ΔmcmA) and Me-Arg285 (ΔmamA). Little is known about the installation and function of these modified residues. The work is solid, novel, and the conclusions are mostly well substantiated. For example, they:
o provided unambiguous mass spectroscopic and crystallographic evidence that McmA is the SAM-dependent methyltransferase responsible for formation of S-methylcysteine
o They provide reasonable evidence supporting their suggestion that cysteine methylation plays an important role in adaptation to a mesophilic lifestyle.
o They provide strong evidence that installation of any of the three modified amino acids (5-methylarginine, thioglycine, or S-methylcysteine) does not affect installation of any of the others, indicating that all these modifications are independently installed.
• I feel that the biggest limitation in the paper is the absence of activity measurements. However, I accept as reasonable their argument that the growth rates are an accurate reflection of the activity, given the current difficulty in obtaining in vitro activities for the M. acetivorans MCR.

We share the reviewers concerns and wish that we were able to perform these analyses; however, a method for isolation of active MCR from *M. acetivorans* is currently lacking. Thus, such experiments are not possible at present.

Specific comments and concerns:
• They generated the ΔmcmA deletion mutant in Methanosarcina acetivorans McrA, purified the MCR and provided unambiguous mass spectroscopic and crystallographic evidence that McmA is the SAM-dependent methyltransferase responsible for formation of S-methylcysteine.

o Their phenotypic analysis of mutants incapable of cysteine methylation suggests that the S -methylcysteine residue plays an important role in adaptation to a mesophilic lifestyle. Furthermore, they observed a 30% slower growth rate on dimethyl sulfide and 12% decrease in growth yield on TMA. Oddly, ΔmcmA grows faster and with higher yield at higher temperatures (42 oC) than wild type.

• To examine the interactions between the S -methylcysteine residue (mcmA) and the previously characterized thioglycine and 5-methylarginine modifications, they generated M. acetivorans mutants lacking the three known modification genes in all possible combinations.

o They unambiguously showed by MS approaches that the installation of any of the three modified amino acids (5-methylarginine, thioglycine, or S-methylcysteine) does not affect installation of any of the others, indicating that all these modifications are independently installed.
• From before: neither thioglycine or methylarginine is essential for methanogenic growth of Methanosarcina acetivorans. Nevertheless, mutants lacking these genes display severe growth defect on substrates with low free energy yields (such as dimethyl sulfide or acetate) or when the cells are grown under stressful conditions (such as elevated temperatures or oxidative stress).
o They state that their phenotypic analyses revealed “complex, physiologically relevant interactions between the modified residues, which alter the thermal stability of MCR in a combinatorial fashion that is not readily predictable from the phenotypes of single mutants”.

♣ The in vitro thermofluor assays appear to be used to gauge interactions between the modified residues and to bring a better understanding of the effects observed in the growth rate and yield. Thus, in terms of reporting on interactions, I would expect that the in vitro temperature dependence would reflect the temp dependence of the growth rate/yield. The relationship between the substrate-dependence of the growth rate/yield and the thermofluor assay seems much less intuitive, especially since these two parameters (substrate dependence and temp dependence of growth rate doesn’t overlap very well).

The reviewer is correct: one might expect that the in vitro temperature dependence would reflect the temp dependence of the growth rate/yield. Thus, the failure to observe this linkage (in most cases) is surprising. However, as we point out in the paper, ***the fact that this linkage is not observed is informative, rather than problematic.*** Our reasoning for conducting both growth and in vitro stability assays is as follows:

MCR comprises ~10% of the cell protein and is widely hypothesized to catalyze the rate-limiting step during methanogenic growth. Thus, mutations that negatively effect turnover of the enzyme may affect growth rate and cell yield. Thus, if the loss of modified residues diminished the turnover rate of MCR in the cell, this should be reflected in the growth rate and/or growth yield.

Significantly, other factors can also affect growth rate and yield. In particular, changes that affect enzyme stability will also affect growth rate and yield due to the need to replenish active enzyme at higher rates than the wild type. Thus, establishing the stability of the unmodified MCR derivatives via the Thermofluor assay allows us to distinguish between changes in catalysis and changes in protein stability.

Accordingly, when the in vitro thermal stability data correlate with the growth data, we conclude that the modified amino acids impact the global stability of the enzyme complex. When the in vitro thermal stability data do not correlate with the growth data, we conclude that the loss of these amino acid modifications affects catalysis. ***We have attempted to make these points more clearly in the revised manuscript.***

♣ The suggestion that “the S-methylcysteine modification is involved in the adaptation of MCR to mesophilic environments” seems reasonably well substantiated; however, they might note that its occurrence with the nonvariable Thio-Gly is important. Its presence by itself allows it to grow at high temperatures.

We agree with this conclusion and noted this interaction in the original manuscript (also see the following comments).

♣ For example, they show that the ∆ycaO-tfuA/∆mcmA mutant had the most severe growth defect than any single mutant. It also had the most severe growth defect on all substrates tested. The authors suggest that this indicates “that the thioglycine and S-methylcysteine modifications interact synergistically”. However, this interaction does not seem to be reflected in the temperature dependence of the growth rate or growth yield or in the thermofluor assay, which seems inconsistent with the interaction between these residues.

We respectfully disagree on this point, based on the reasoning laid out in the previous response. Thus, we expect the Thermofluor data to be consistent with the growth rate and yield data ***only*** when the loss of the modified residues causes a significant change in the global stability protein.

Our data show that the ∆*mcmA* mutant has faster growth rates than the wild type at higher temperatures, while the ∆*ycaO-tfuA/∆mcmA* double mutant has a slower growth rate. Thus, it is clear that the presence/absence of each modification affects the phenotypes of the other, but not in ways that are easily predicted from the phenotypes of the single mutants (*i.e.* they must interact).

The reviewer is correct to state that the Thermofluor data do not support this interaction, because the melting point of the double mutant is exactly the average of the two single mutants. We interpret this mean that the interaction ***does not*** affect stability, but rather some aspect of enzyme catalysis (as described above).

♣ On the other hand, the thermofluor assays do appear to indicate that interactions between the 5-S-methylarginine and thioglycine modifications influence the thermal stability of MCR. I think that this is consistent with the temperature-dependence of growth rates and yields of the double vs the single mamA deletions and, if so, should be stated. The implications for interactions seem more straightforward for these two residues. If they are both present or both absent, the protein seems more stable and the cells grow faster and to a higher yield.

We agree and have modified the results to highlight that the interaction between 5-S-methylarginine and thioglycine modifications influence the thermal stability of MCR (see lines 260-261). (It is worth pointing out that this observation justifies the need for both the *in vivo* and *in vitro* characterizations of the MCR derivatives.)

♣ I agree with the authors that it does seem that these interactions are a reflection of dynamics not the static structure. It would be really useful if they could look at enzyme activity as a function of temperature. Here the dynamics would most certainly be reflected. Does the activity reflect what is seen in the thermofluor assay? I imagine not. The authors suggest a similar point in discussing the methyl-cysteine modification when they state “Thus, we suspect that the temperature-dependent phenotypes of strains lacking S-methylcysteine are related to catalysis rather than structure”. I think that they might add “static” or “most stable” structure.

We have added the phrase “static structure” as suggested (see line 329).

• What are the activities of the purified variants? It is likely that this will give the most valuable information related to the growth effects. However, the authors do discuss the unfortunate problems with determining activity for the Ma protein using the two assays that have been successful with other systems.

We agree with the reviewer on all points raised in this comment, especially the point regarding the need to assess these biochemically. Unfortunately, obtaining active MCR preparations is notoriously difficult, as noted in several publications (reviewed in Biochemistry. 2019 Apr 5. doi: 10.1021/acs.biochem.9b00164.). To date, we have been unsuccessful in activating the MCR complex from *Methanosarcina acetivorans* *in vitro.* As a result, activity measurements are currently not feasible with the enzyme derived from this organism. We hope that dedicated efforts towards activating the MCR complex from *M. acetivorans* in the near future will overcome this hurdle so that we can address the questions that the reviewer poses here.

We note that the reviewer seems to accept this situation as noted by their comment above: ***“However, I accept as reasonable their argument that the growth rates are an accurate reflection of the activity, given the current difficulty in obtaining in vitro activities for the M. acetivorans MCR.”***

---------------

Reviewer #3:
The manuscript by Nayak et al. details a thorough investigation into the functional interactions of three post-translational modifications (PTMs) found in the active site region of methyl-coenzyme M reductase (MCR) from Methanosarcina acetivorans. CRISPR-Cas9 was used to knock-out the genes required for the installation of the thioglycine, 5-(S)-methylarginine, and/or S-methylcysteine residues (the latter of which was previously unreported) in all possible combinations. The resulting strains were then tested for their growth rates and yields on a variety of methanogenic substrates. The MCR variants were generated with tandem affinity purification (TAP)-tags on the N-terminus of each McrG subunit and the melting temperatures and high-resolution crystal structures of the purified enzymes were determined. Together the data suggest that the PTMs are not essential, with little to no influence on the structure of the enzyme and its active site. However, the PTMs seem to have unanticipated epistatic effects on the thermal stability and/or dynamics of the enzyme, with the S-methylcysteine residue possibly playing a role in the adaptation of methanogens to a mesophilic lifestyle (consistent with its phylogenetic distribution). In all, this is a solid and significant work that is worthy of publication in PLOS Biology pending some minor revisions.

1. Figure 5. It is unclear why the average melting temperature/standard deviation of wild-type MCR should be different in each of the panels A, B, and C. 2.

We thank the reviewer for their comments. In our experience, the MCR complex is stable at room temperature (under aerobic conditions) for ~24-36 hours post-purification (and the stability decreases as the temperature). To ensure that the protein complex retains its native (albeit inactive) conformation, the thermal stability assays were conducted within 1-3 hours of protein purification. Given the instability of the protein and the large culture volumes needed for protein purification it was extremely cumbersome to purify the eight different protein variants at once. Therefore, protein purification for this study was conducted in three batches, with the wild type included each day to control for the small variations noted by the reviewer. The three panels in the figure indicate these batches. **We have added a statement in the legend of Figure 5 to clarify this point to the readers.**

We wish to stress that the day to day differences in the thermal stability of WT MCR was very small (< 1^o^C). We suspect that these minor differences are due to slight variations in temperature during purification and in the time required to set up and perform the thermal stability assays after purification.

A few minor typos were noticed throughout that should be corrected in the final version.

We have scanned the latest draft of the manuscript for typos and have corrected all the ones we could find.

---------------


Reviewer #4:
The paper by Nayak et al. identifies the locus MA4551 as necessary for the methylation of cysteine during the posttranslational modification of Mcr. It further examines the deletion of the thioglycine and methylarginine PTMs in all possible combinations with the deletion of methylcysteinyl PTM. In the absence of direct enzyme assays which are not technically possible, the phenotype is inferred from growth assays, and the effects on Mcr structure are inferred from crystal structures. In some respects, this is an impressive demonstration of technology.

However, the major caveat to the work is that the mutations were introduced into the genome. Because the Mcr is an essential gene, the mutant is under selection as soon as it is formed, and no tests were performed for secondary mutations that affect the phenotype. To convincingly demonstrate the phenotypes of the mcmA and mamA deletions, the genes should be complemented and restoration of the wild-type phenotype shown. Alternatively, the mutants could be resequenced and shown to be free of secondary mutations.

We have performed complete genome resequencing for each of the mutants and compared them to the parental strain as requested by the reviewer. Although a few secondary mutations were observed, none were specifically correlated with any of the mutations that we introduced. Instead, the secondary mutations we observed were correlated with the lineage from which they were derived. Thus, it is highly likely that these secondary mutations were not selected as ones that suppress the phenotype of the introduced mutations. **This is now described in the text of the revised paper (lines 478-491) and** **shown in a revised Figure S3.**

Line 31, 253-254, and 312-319. The suggestion that McmA is involved in adaptation to mesophilic growth is not well supported. Although not significant, the mcmA deletion mutant also grew better than WT at low temperature. Likewise, the gene is common in many thermophilic methanogens, such as Methanothermus and Methanothermobacter (Fig. 2), and absent in a number of mesophiles in addition to Methanomassiliicoccus.

We respectfully disagree on this point. The fact that the *S-*methylcysteine modification helps with growth at low temperatures does not mean that it is required. Indeed, our data show that the mutant lacking this modification grows reasonably well, just not as well as the parent. The potential mesophilic advantage could also be conferred by other mechanisms, thus the phylogenetic distribution of the *mcmA* gene does not have to be perfectly correlated with growth temperature for our hypothesis to be correct. **Nevertheless, we concede that this idea is currently speculative. With this in mind, we have substantially softened our wording to indicate this is a speculative conclusion** (see lines 31-32 and lines 320-323).

How was the nature of the mutations verified? Evidence should be provided that the mutants were free of wild-type and have the stated sequence. Lines 350-353.

See note re: Illumina resequencing above.

If the Mcr is rate-limiting during growth, the function of the enzyme should be measurable from the rates of methanogenesis by resting cells. Why wasn’t this done?

We do not believe that rates of methanogenesis in resting cells will be informative in this case, because it is possible (and we believe likely) that these rates will not accurately reflect the rate of MCR catalysis. This is because in methanogenesis is directly coupled to generation of an ion-motive force across the cell membrane. This ion motive force is used to drive synthesis of ATP, which is continuously consumed by the cellular process required for growth and maintenance. If consumption of the ion-motive force via ATP synthesis is faster than methane production (which is implied by the assumption that MCR is the rate-limiting step in methanogenesis), then measurement of methane production during active growth rate will reflect activity of the enzyme (as implied by the reviewer). However, ***resting cells do not consume ATP*** (other than for maintenance). Thus, the ion motive force is not dissipated and instead produces a “backpressure” on each of the energy-conserving steps in the methanogenic pathway. In this situation, MCR is unlikely to be the rate-limiting step in methane production. Instead, it seems much more likely that spontaneous dissipation of the ion-motive force is the rate-limiting step in the process. Given the uncertainties inherent to this situation, we do not feel that the labor-intensive process of cell suspension assays is merited.

“genes that install”, “installation of this modification” throughout. This terminology is cumbersome and misleading. These genes encode enzymes that catalyze chemical reactions. “Install” is poor terminology.

We have made the modifications per the reviewer’s request

Define “TAP-tagged” at first mention.

We have provided this definition (see lines 204-205) in the text.

Supplementary materials. Accept track changes in document.

We have made the modifications per the reviewer’s request (see supplement)

How were the growth rates calculated? Was a correction made for the large inoculum size?

Yes a correction was made for the large inoculum size. 1 mL culture was added to 9 mL HS medium so that the final volume was 10 mL. Growth rate was determined for three biological replicates (i.e. three cultures inoculated with three independent colonies of the mutant strain) that were acclimated to the growth conditions for at least 5-10 generations. Growth rate measurements were performed by measuring the optical density at a wavelength of 600 nm using a spectronic 200E (Thermofisher Scientific) spectrophotometer outfitted with a test tube holder that would fit a Balch tube. Optical density (OD) readings were only taken in the linear range of the spectrophotometer (0.02-1.5) and dilutions were performed as necessary. OD readings were used to plot a growth curve. A semi-log plot of the growth curve was then used to calculate the growth rate by identifying the set of points (a minimum of five) with the best fit to a linear regression model with an R^2^ threshold greater than 0.995. The average of three independent growth rates was designated as the mean growth rate for a given strain for a given growth condition. **We have added this information to the materials and methods section (see lines 501-511).**


Growth yields. How were the growth yields measured? The reported absorbances are >3, which is outside the linear range of most spectrophotometers.

Yes, the reviewer is indeed correct that the final yield was often beyond the range reported by our spectrophotometer. In these cases, a dilution curve was performed such that the OD reading was within the linear range of the instrument (see above). **We have clarified this in the materials and methods section (see lines 501-511).**

Tables S2-S13. Overall, the formatting on these tables made them difficult to read. Please reformat. Use consistent coloring for significance for p-values. Compare S11 to the others.

We have reformatted the table (see supplement)

Figure 5. The lines are not clear with the colors and symbols chosen. Please chose different colors.

We have increased the line thickness to accommodate the reviewer’s request.
