# Supplementary material for: Functional interactions between posttranslationally modified amino acids of methyl-coenzyme M reductase in Methanosarcina acetivorans
Source: PLoS Biol. 2020 Feb 24;18(2):e3000507. doi: 10.1371/journal.pbio.3000507 (PMC7058361; doi:10.1371/journal.pbio.3000507)
Supplement: Attachment — Submitted filename: response to reviews.docx [file pbio.3000507.s038.docx]

**Point-by-point response to the reviews of PBIOLOGY-D-19-02599R1**

We have attempted to revise all the files as requested by your staff.
